# Deep immune profiling of endometrial and peripheral blood cells in endometriosis

**DOI:** 10.1093/humrep/deag090

**Published:** 2026-06-05

**Authors:** A Kisovar, M Buttenschoen, Q Obrigewitch, K Powell, P Klenerman, K Zondervan, C M Becker, I E Granne, J H Southcombe

**Affiliations:** Nuffield Department of Women’s and Reproductive Health, University of Oxford, L3 Women’s Centre, John Radcliffe Hospital, Oxford, UK; Department of Statistics, University of Oxford, Oxford, UK; Nuffield Department of Women’s and Reproductive Health, University of Oxford, L3 Women’s Centre, John Radcliffe Hospital, Oxford, UK; Peter Medawar Building for Pathogen Research/Translational Gastroenterology Unit, University of Oxford, Oxford, UK; Peter Medawar Building for Pathogen Research/Translational Gastroenterology Unit, University of Oxford, Oxford, UK; Nuffield Department of Women’s and Reproductive Health, University of Oxford, L3 Women’s Centre, John Radcliffe Hospital, Oxford, UK; Nuffield Department of Women’s and Reproductive Health, University of Oxford, L3 Women’s Centre, John Radcliffe Hospital, Oxford, UK; Nuffield Department of Women’s and Reproductive Health, University of Oxford, L3 Women’s Centre, John Radcliffe Hospital, Oxford, UK; Nuffield Department of Women’s and Reproductive Health, University of Oxford, L3 Women’s Centre, John Radcliffe Hospital, Oxford, UK

**Keywords:** endometrium, immunity, endometriosis, menstrual cycle, spectral flow cytometry

## Abstract

**STUDY QUESTION:**

How is endometrial and systemic immunity modulated throughout the menstrual cycle and are there changes in women with endometriosis?

**SUMMARY ANSWER:**

Endometriosis is associated with reduced endometrial early natural killer (NK) cells and increased mucosal-associated invariant T (MAIT)-like CD8+ T cells, with cyclical variation.

**WHAT IS KNOWN ALREADY:**

The endometrial mucosa contains innate and adaptive immune cells that fluctuate across the menstrual cycle. Immune dysregulation is found in endometriosis, however few studies have broadly assessed endometrial immune single-cell proteome phenotypes.

**STUDY DESIGN, SIZE, DURATION:**

This observational cross-sectional immune phenotyping study included 40 participants (28 with surgically confirmed endometriosis and 12 controls).

**PARTICIPANTS/MATERIALS, SETTING, METHODS:**

Endometrial and peripheral blood samples were analysed by spectral flow cytometry using a 36-parameter immune phenotyping panel and a 13-parameter MAIT-specific panel. Totals of 1 950 292 circulating and 1 023 215 endometrial immune cells were profiled. Full-thickness uterine biopsies (n = 3) underwent multiplex immunohistochemical imaging to assess spatial organization across the menstrual cycle.

**MAIN RESULTS AND THE ROLE OF CHANCE:**

Compared with controls, patients with endometriosis exhibited decreased endometrial early NK cells (*P*.adj = 0.006, log2FC = −1.369) and increased MAIT-like CD8+ T cells (CD161+CD8+CD3+) (*P*.adj = 0.033, log2FC = 1.415). The MAIT cells (CD161+Va7.2+CD3+) peaked during ovulation and the implantation window (*P*.adj < 0.05). Peripheral immunity also showed cyclical variation with increased early NK cells (*P*.adj = 0.001, log2FC = 1.052) and decreased effector CD4 T (*P*.adj = 0.002/log2FC = −2.010) and effector CD8 T cells (*P*.adj = 0.002, log2FC = −1.180) in the endometriosis group.

**LIMITATIONS, REASONS FOR CAUTION:**

The cytometric panel design was biased towards acquired immunity, and the endometriosis patient sample size prevented subtype analysis.

**WIDER IMPLICATIONS OF THE FINDINGS:**

MAIT cell dysregulation represents a novel feature of endometriosis, potentially contributing to subfertility and providing new avenues for therapeutic development.

**STUDY FUNDING/COMPETING INTEREST(S):**

This study was funded by the Nuffield Department of Women’s and Reproductive Health, University of Oxford, and also supported from the University of Oxford Medical Sciences HIDI Internal Fund Award (0010398), Academy of Medical Science Award (SBF007\100078) and, British Society for Immunology Career Enhancing Grant. C.M.B. has a consultancy role with ObsEva, Theramex, Roche Diagnostics, Sumitovant, Gedeon Richter, Gesyntha, and they have Research Grants from Bayer, Gesyntha, and Serac Life Services. K.Z. is a Board member (non-remunerated) of the World Endometriosis Research Foundation. She has consultancy status with Roche Diagnostics and Gedeon Richter. The remaining authors declare that the research was conducted in the absence of any commercial or financial relationships that could be construed as a potential conflict of interest.

**TRIAL REGISTRATION NUMBER:**

N/A.

## Introduction

Endometriosis is a common disease affecting women defined by the presence of endometrium-like tissue outside of the uterus (Zondervan *et al.*, 2018). It affects 1 in 10 women, mostly of reproductive age, equivalent to ∼190 million women worldwide (Zondervan *et al.*, 2020). Using revised criteria developed by the American Fertility Society and American Society of Reproductive Medicine (rAFS/ASRM), endometriosis can be divided into four stages considering lesion size, location, and extent of adhesions. However, this does not necessarily correlate with the severity of associated conditions, namely chronic pelvic pain and subfertility (Prescott *et al.*, 2016; Coxon *et al.*, 2023).

The most common cause for endometriosis-associated subfertility is adhesions, which can affect the uterus, uterine tubes, and ovaries disrupting their normal reproductive functions (Bonavina and Taylor, 2022). However, considering that the operative treatment does not improve pregnancy rates in endometriosis patients undergoing ART, altered endometrial immunity influencing endometrial receptivity has been suggested as one of the additional factors leading to endometriosis-associated subfertility (de Ziegler *et al.*, 2010; Dunselman *et al.*, 2014; Lessey and Kim, 2017). In addition, several immunological disorders are linked to reproductive failure, such as anti-phospholipid syndrome and autoimmune thyroid diseases (Deroux *et al.*, 2017). Women with endometriosis also have an increased risk of autoimmune disorders (Shigesi *et al.*, 2025), nevertheless, it is unclear how systemic autoimmune disorders influence the endometrium. This study aims to explore peripheral blood and endometrial immune cells in women with endometriosis.

The endometrium, comprised of stroma, epithelium, immune, and vascular cells, undergoes hormone-regulated cyclical changes necessary to facilitate and sustain embryo implantation. Disruptions to cyclical structural and immune changes are implicated in a variety of reproductive disorders, including infertility, implantation failure, and miscarriage (Gellersen and Brosens, 2014). Endometrial mucosa is populated by various immune cells, which account for 5–30% of total endometrial cells across the cycle (Mareckova *et al.*, 2024). During the menstrual phase, shedding of the endometrial lining triggers the release of proinflammatory cytokines and the influx of immune cells, such as macrophages and neutrophils, to remove tissue debris and pathogens. In the proliferative phase, T cells are the most abundant leukocytes, comprising 40–60% of all endometrial leukocytes. While their absolute numbers remain relatively constant, their proportion decreases to < 10% in the late secretory phase due to the accumulation of endometrial natural killer (NK) cells postovulation (Wira *et al.*, 2015). During the window of embryonic implantation (WOI), the immune system undergoes temporary modifications to facilitate embryo attachment and invasion of trophoblast cells from the developing conceptus, which expresses paternal alloantigens that could trigger an inflammatory response detrimental to reproductive success (Robertson *et al.*, 2018). During the WOI, oestrogen and progesterone enhance luminal innate cells specialized in tissue remodelling and suppress CD8+ T (CD8 T) and NK cell cytotoxicity, providing a tolerogenic environment that effectively prevents T cell-mediated allograft rejection (Schumacher *et al.*, 2018).

In endometriosis, evidence indicates changes to endometrial NK cells and macrophages (Wira *et al.*, 2015; Zhang *et al.*, 2022), but comprehensive data on other immune subtypes and their role in endometriosis are scarce (Ahmed *et al.*, 2025). This study was aimed to utilize high-parameter spectral flow cytometry to broadly define immune cell types using a pre-established protocol for peripheral blood immune cell analysis (Park *et al.*, 2020) with modifications to include cellular proteins relevant to tissue residency (CD103/CD69) (Gray and Farber, 2022). We investigated conventional NK and T cell populations with a focus on their memory status and phenotypic characteristics. We also examined immune cell subsets, implicated in pregnancy and its complications, yet underexplored in endometriosis, NK T cells (NKT), mucosal-associated invariant T (MAIT), dendritic, and B cells (Ahmed *et al.*, 2025). We aimed to characterize endometrial immune cell populations, comparing cell proportions across the menstrual cycle. In addition, we investigated cellular phenotypes comparing endometriosis versus controls in both endometrial and systemic immunity.

## Materials and methods

### Study design and ethical approval

Females of reproductive age (18–39 years) with regular uterine cycles at least 3-month postmiscarriage or hormonal treatment were eligible for the study. Participants for the deep immune phenotyping study were undergoing surgical intervention for endometriosis diagnosis and/or treatment at the Endometriosis CaRe Centre or were recruited for research purposes through the Subfertility Clinic, both situated in the John Radcliffe Hospital, University of Oxford. Patients had no other gynaecological pathologies other than being classified subfertile or <3 miscarriages experienced; PCOS, hydrosalpinx, pelvic inflammatory disease, cancer or diagnosis with a known factor associated with recurrent pregnancy loss were exclusion criteria. They were recruited into two prospective cross-sectional studies: FENOX (Research Ethics Committee (REC) Ref: 17/SC/0664) and The PIP (Peri-implantation ImmunoProfiling) study (REC ref: 18/SC/0216).

In addition, three full-thickness endometrial biopsies from two endometriosis patients in the proliferative phase and non-endometriosis control from the secretory phase were selected from our tissue bank and underwent multiplex immunohistochemical analysis. Patients were recruited into two studies (REC reference: 19/LO/1802): Investigation of gene expression in normal endometrium and endometriosis COREC 00.156 and Investigating the causes of infertility OxREC No.C02.358. In all cases, informed written consent was obtained.

### Deep immune phenotyping: sample collection and processing

**Figure 1.**
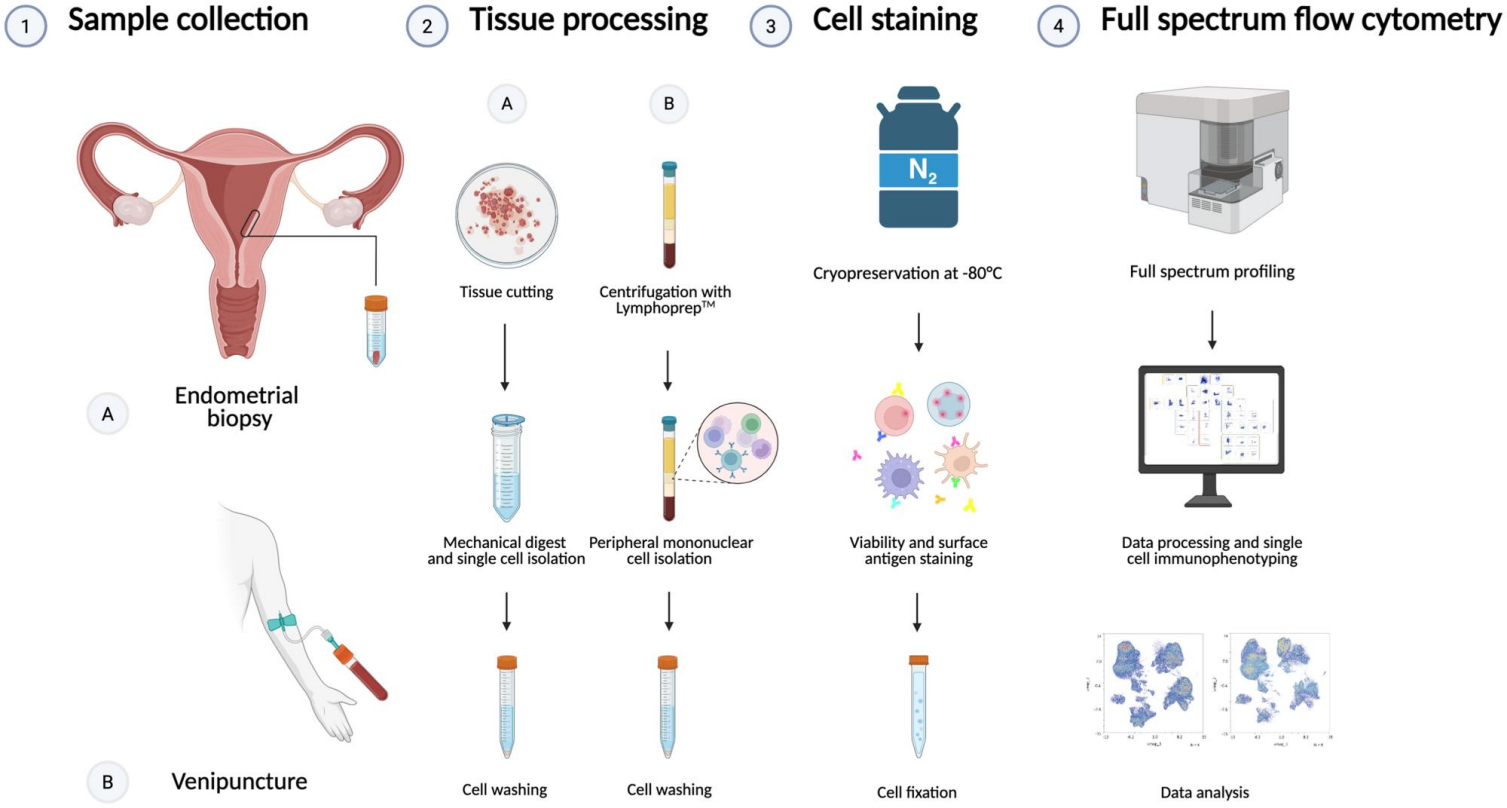
**Sample workup for full-spectrum flow cytometry analysis.** Endometrial and peripheral blood samples were processed following standard protocols within 6 h of collection. Endometrial tissue was mechanically digested into single-cell suspensions and blood mononuclear cells were isolated via Lymphoprep™ density gradient centrifugation. Samples were then stored in liquid nitrogen until cell staining and data acquisition.

Peripheral blood samples were acquired before surgery for endometriosis or during clinical examination in the miscarriage clinic into 4.5 ml/9 ml Lithium/Sodium Heparin vacutainers (BD Biosciences, UK). Endometrial biopsies were collected using an Endocell^®^ disposable cell sampler (Cooper Surgical, USA) during surgery for endometriosis or at the recruitment visit for the PIP study. All samples were obtained according to the manufacturer’s instructions and processed within 6 h from collection. Peripheral blood mononuclear cells (PBMC) were isolated from whole blood by density gradient centrifugation using Lymphoprep™ (Gen Group, USA). Endometrial tissue was mechanically digested in digest media (Iscove’s Modified Dulbecco’s medium supplemented with 10% FCS, 1% Human Serum, and 1% Penicillin/Streptomycin/Glutamine (All Sigma, USA)) and passed through sequential 70 and 40 µm cell strainers to obtain a human endometrial single cell (HEC) suspension, as previously described (Granne *et al.*, 2022). All single-cell suspensions were stored in freezing media (10% DMSO/FCS) in liquid nitrogen until cell staining was performed. The sample workup is presented in Fig. 1.

### Spectral flow cytometry panel design for deep immune cell phenotyping

In collaboration with Cytek and the Nuffield Department of Experimental Medicine, University of Oxford, a 36-parameter panel was developed focusing on adaptive immune subtypes (Table 1). The development of the panel involved selecting an optimal combination of 36 fluorochromes based on specific criteria, including unique spectral signature (similarity index) and overall fluorochrome compatibility (complexity index) (Supplementary Figs S1 and S2). Following a pre-determined spectral flow cytometry protocol with modifications, optimal titres for 13 (CD103, CD11b, CD11c, CD161, CD24, CD25, CD4, CD45RA, CD57, CD69, CD8, CXCR3, HLA DR, and Zombie-NIR) out of 36 reagents from our spectral flow panel for both HEC and PBMC based on the median fluorescence intensity were determined, following principles for panel testing advised by Cytek (Park *et al.*, 2020). Spectral unmixing accuracy, marker resolution, steric hindrance, and spread impact on the resolution were assessed before fine-tuning of the staining protocol and data acquisition. As a proof of concept, following the same panel design approach, cell staining, and data analysis steps, we designed a validation study using TCR Vα7.2 in combination with other relevant markers shown in Table 2 to delineate MAIT cells in patients with endometriosis.

**Table 1.** Reagents used for deep immune phenotyping with full-spectrum flow cytometry.

Marker	Target cell types	Clone	Fluorochrome
**Viability**	Viable cells		Zombie NIR
**CD45**	Leukocytes	HI130	PerCP
**CD3**	T cells, NKT-like cells	SK7	BV510
**CD4**	CD4 T and NKT-like cells	SK3	cFluor^®^ B532
**CD8**	CD8 T, NK, and NKT-like cells	SK1	cFluor^®^ V610
**CD25**	Regulatory T cells	BC96	PE-Cy5
**TCRγδ**	γδ T cells	B1.1	PerCP-eFluor 710
**CD14**	Monocyte differentiation	63D3	Spark Blue 550
**CD16**	Monocyte, NK cell, and dendritic cell differentiation	3G8	BUV496
**CD11c**	Dendritic cell differentiation	B-ly6	BUV661
**CD11b**	Monocyte/macrophages, granulocytes, and NK cells	ICRF44	PerCP-Cy5.5
**CD19**	B cells	HIB19	Spark NIR 685
**CD20**	B cells	HI47	Pacific Orange
**CD24**	B cell differentiation	SN3	PE-eFluor 610
**IgD**	B cell differentiation	IA6-2	BV480
**IgG**	B cell differentiation	G18.145	BV605
**IgM**	B cell differentiation	MHM-88	BV570
**CD1c**	Dendritic cells, NKT-like cells	L161	Alexa Fluor 647
**CD123 (IL3R)**	Plasmacytoid dendritic cells	6H6	Super Bright 436
**CD56**	NK cells, γδ T cell activation	NCAM16.2	BUV737
**CD197 (CCR7)**	T cell differentiation	G043H7	BV421
**CD27**	T and B cell differentiation	O323	APC
**CD28**	T cell and NK cell differentiation	CD28.2	BV650
**CD45RA**	T cell and dendritic cell differentiation	5H9	BUV395
**CD127**	Cytokine receptor; T cell differentiation	HIL-7R-M21	APC-R700
**CD196 (CCR6)**	Chemokine receptor; T cell and B cell differentiation	g034e3	BV711
**CD195 (CCR5)**	Chemokine receptor; monocyte, dendritic cell, T cell, and B cell differentiation	2D7/CCR5	BUV563
**CD185 (CXCR5)**	Chemokine receptor; T cell differentiation	RF8B2	BV750
**CD183 (CXCR3)**	Chemokine receptor; dendritic cell, T cell, and B cell differentiation	G025H7	PE
**HLA DR**	T cell and monocyte activation, NK cell lineage discrimination, dendritic cell lineage marker	L243	APC-eFluor 780
**CD38**	Monocyte, dendritic cell, T cell, and B cell activation/differentiation	HIT2	APC/Fire 810
**CD57**	NK and CD8+ T cell immune senescence	NK-1	BB515
**CD161 (KLRB1)**	NK cell cytotoxicity and T cells	HP-3G10	eFluor 450
**CD279 (PD1)**	T cell inhibitory receptor	EH12.2H7	BV785
**CD69**	Tissue residency	FN50	PE-Cy7
**CD103**	Tissue residency	Ber-ACT8	BUV805

**Table 2.** Reagents used for the validation immune phenotyping study of MAIT cells with full-spectrum flow cytometry.

Marker	Target cell types	Clone	Fluorochrome
**Viability**	Viable cells		Zombie Aqua
**CD3**	T cells, NKT-Like cells	SK7	Alexa Fluor 700
**CD4**	CD4 T and NKT-like cells	RPA-T4	BV711
**CD8**	CD8 T, NK, and NKT-like cells	SK1	eFluor™ B515
**CD45RA**	T cell and dendritic cell differentiation	HI100	eFluor™ 506
**CD197 (CCR7)**	T cell differentiation	G043H7	eFluor™ BYG575
**CD27**	T and B cell differentiation	OAT48	eFluor™ R840
**CD127**	Cytokine receptor; T cell differentiation	A019D5	eFluor™ 659
**CD161 (KLRB1)**	NK cell cytotoxicity and T cells	HP-3G10	eFluor™ 450
**TCR Va7.2**	MAIT cells	3C10	BV785
**CD279 (PD1)**	T cell inhibitory receptor	EH12.2H7	PE/Cyanine 7
**CD69**	Tissue residency	FN50	BV605
**CD103**	Tissue residency	Ber-ACT8	PE/Dazzle 594

### Immune cell staining for flow cytometry

After thawing in complete RPMI media (RPMI 1640 supplemented with 10% FCS, 2 mM glutamine, 100 IU/ml penicillin, and 100 µg/ml streptomycin), cells were washed by centrifugation (300 *g*) and incubated with 100 ml Zombie NIR viability dye (Biolegend) (1:1000 dilution in phosphate-buffered saline (PBS)) for 20 min at room temperature in the dark and then washed with PBS. Next 2×10^6^ cells were pelleted and 10 µl Brilliant Stain Buffer Plus (BD Bioscience) then 5 µl True-Stain Monocyte Blocker (Biolegend) were added with vortexing between reagents. Antibodies were diluted at validated concentrations (Supplementary Table S1) in Cell Staining Buffer (Biolegend, USA). Certain antibodies were added sequentially with vortexing at each step before applying the multi-antibody mix to minimize steric hindrance, namely CXCR5, CCR6, CCR7, and CCR5 followed by 10 min incubation, next TCRγδ, IgD, and PD-1 followed by 10 min incubation, next CD20, CD161, and CD28 followed by 10 min incubation, then the remaining antibodies were added with incubation for a further 30 min. All incubations were on ice in the dark. Finally, all samples were washed in Cell Staining Buffer and fixed with FluoroFix™ Buffer (Biolegend, USA) following manufacturer instructions before data acquisition. A full protocol is available in the Supplementary Materials and Methods. Minor modifications were made to the protocol for the MAIT validation panel: 100 µl Zombie Aqua viability dye (1:1000 dilution, Biolegend) was substituted and 2.5 µl of each antibody was used to stain cells.

### Flow cytometry data acquisition

Data for the deep-immune panel were acquired with Cytek^®^ Aurora system (5-laser 16UV-16V-14B-10YG-8R; Cytek Biosciences, USA), the MAIT validation panel was acquired using Cytek^®^ Northern Lights (3-laser V-B-R; Cytek Biosciences, USA), both using SpectroFlo^®^ software. To minimize batch effects, all data were collected within 1 week. Before data acquisition, instrument performance was ensured with quality control beads. We maintained consistency by employing the same event rate and threshold settings throughout the data acquisition process. Additionally, identical single stain controls (reference controls) were used for live unmixing of the samples.

### Flow cytometry data processing

Raw high-dimensional flow cytometry data from all endometrial and peripheral blood samples were processed with the OmiqM (Dotmatics, USA) platform. To enhance data quality, we first applied the flowAI tool, which corrects baseline fluorescence shifts, ensures uniform data acquisition by addressing flow rate variations, and minimizes fluorescence spillover between channels to enhance data quality (Monaco *et al.*, 2016). This was followed by excluding debris and non-single cell events by utilizing forward scatter (FSC) and side scatter (SSC) parameters and then gating on live (Zombie NIR-) immune cells (CD45+). To achieve data comparability across samples, we performed normalization with fdaNorm for 31 parameters targeted to one of the peripheral blood samples. Then, the normalization process was reapplied for the three remaining parameters highly expressed in the endometrium (CD69, CD183, CD56) and the baseline was set to one of the endometrial samples (Hahne *et al.*, 2010). Unmixing accuracy for CD45+ events was assessed before and after normalization by examining Nx1 and NxN plots with minimal compensation adjustments before normalization.

### Flow cytometry data analysis

Patients’ characteristics with potential immunomodulatory effects (BMI and age) were compared between the two analysis groups. The normality of continuous variables within each group was assessed using the Shapiro–Wilk test. Normally distributed variables were compared using independent samples *t*-tests, while non-normally distributed variables were compared using the Wilcoxon rank-sum test. Pre-processed multiparameter flow cytometry data were first analysed with OmiqM (Dotmatics, USA) platform. Analysis of CD45+ cells was first performed on matched endometrial and peripheral blood samples. Next, we investigated all endometrial and peripheral blood CD45+ cells separately, first assessing potential immune modulating factors such as age, BMI, parity, history of miscarriage, and menstrual cycle, then comparing endometriosis patients with non-endometriosis controls. Data were analysed using standard objective gating strategies (Supplementary Fig. S3) or a non-linear dimensionality reduction technique, i.e. uniform manifold approximation and projection (UMAP), was applied before clustering to visualize the diverse cell populations within our dataset (McInnes *et al.*, 2018). We then performed unsupervised clustering with FlowSOM based on lineage markers and visualized meta-clusters using UMAP (Van Gassen *et al.*, 2015). After cluster annotation with a conventional clustered heatmap analysis, the data were exported and further analysed in R Studio (R version 4.3.1). We identified and mitigated confounding variables using the sva package (3.48.0) before the DESeq2 package (1.40.2) was used to calculate differences in meta-cluster cell counts between different groups of interest using a model based on the negative binomial distribution (Leek *et al.*, 2012; Love *et al.*, 2014). Outcomes with *P*.adj < 0.05 and |log2FC|>1 were assigned as statistically significant. For the differential analysis of marker expressions, one-way ANOVA on ranks test from stats package (4.4.0) was used to assess if patient groups and controls share the same distribution, with 1088 tests conducted for 34 markers and 31 cell clusters. Adjusted *P*-values were calculated with the Benjamini–Hochberg procedure for all tested comparisons.

### Immune cell staining using multiplex immunohistochemistry

Multiplex immunohistochemistry with ZellScannerONE™ ChipCytometry™ followed an established protocol (Fitzpatrick *et al.*, 2021). OCT-embedded endometrial samples were cryosectioned onto coverslips and inserted in cytometer chips (Zellsafe Tissue chips, Zellkraftwerk, Germany). Sections were fixed for 10 min at room temperature with 4% paraformaldehyde before being rinsed with 10 ml PBS. Non-specific binding was inhibited by incubation in 5% goat serum in PBS at room temperature for 1 h. For staining, fluorophore-conjugated antibodies were diluted in PBS. Immunostaining was performed with up to three colours used simultaneously, and the sample was repeatedly photobleached after the image had been taken. Antibodies used were: 1.5 µg CD4-PE (REA623; Miltenyi), 5.5 µg CD8-PerCP/Cy5.5 (SK1; Biolegend), 8 µg CD14-PE (599; Miltenyi), 4 µg CD19-PE (REA675; Miltenyi), 8 µg CD20-PE (LT20; Miltenyi); 8 µg CD26-PE (FR10-11G9; Miltenyi), 1 µg CD31-PerCP/Cy5.5 (WM59; Biolegend), 8 µg CD56-FITC (REA196; Miltenyi), 8 µg CD68-AF488 (KP1; Santa Cruz), 4 µg CD69-PE (REA824; Miltenyi), 10 µg CD103-PE (Ber-ACT8; Biolegend), 1 µg Pan-cytokeratin-FITC (C-11; GeneTex), 1 µg SMA-FITC (REA650; Miltenyi), and 6 µg CD161-PE (REA631; Miltenyi). The steps were repeated as many times as needed to complete the panel. Images were captured using a ZellScannerONE™ ChipCytometry™ instrument (Canopy Biosciences, USA) and ZellExplorer software (Canopy Biosciences, USA).

## Results

Patient metadata from the different studies included in this paper are available in Supplementary Tables S2, S3 and S4.

### Deep phenotyping of immune cells

There were 32 study participants included in two analysis groups endometriosis (n = 20) and control (n = 12), as shown in Table 3: ‘All participants’. Patients in both groups had similar ages (*P* = 0.915) and while endometriosis patients have a lower BMI this was not significantly different (*P* = 0.08). Menstrual cycle phase was recorded per quarter (calculated as the proportion of total cycle length with an additional sample group in the WOI, where the LH surge was known and this could be determined), along with parity, known subfertility, and rASRM staging information. Single immune cells were isolated from endometrial or peripheral blood samples and subjected to full-spectrum flow cytometry (Fig. 1). We designed a flow cytometry panel to broadly assess immune cell phenotypes (including subtypes of T, NK, B, monocytes, dendritic cells) with additional phenotypic activation/functional features. The panel was adapted from Park *et al.* (2020) to include additional markers known to be important in tissue residency in the endometrium (Southcombe *et al.*, 2017; Granne *et al.*, 2022). The full panel of antibodies to delineate immune cell populations and the target cell types/function for each marker is shown in Table 1.

**Table 3.** Patient characteristics from all three analysis groups.

	All participants		Case:control cohort		Validation
	**Endometriosis** (n = 20)	**Control** (n = 12)	**Endometriosis** (n = 19)	**Control** (n = 8)	**Endometriosis** (n = 8)
**Age**	33 ± 5	32 ± 6	33 ± 5	32 ± 6	36 ± 7
**BMI**	23 ± 7	29.3 ± 6	23 ± 7	29 ± 5	24 ± 6
**Cycle quarter**					
1	3	1	2	1	2
2	4	1	4	1	1
3	6	0	6	0	1
3.5 (WOI)	3	10	3	6	2
4	4	0	4	0	2
**rASRM stage**					
I/II	11	N/A	12	N/A	N/A
III/IV	8	N/A	8	N/A	N/A
**Parous**	1	3	8	2	N/A
**Previous miscarriage**	6	9	6	5	N/A
**Known subfertility**	4	0	4	0	2
**Endometrial sample**	18	12	17	8	8
**Peripheral blood sample**	15	12	15	8	N/A
**Endometrial CD45+ cells**	615 475	267 706	615 475	246 321	140 034
**Circulating CD45+ cells**	1 111 110	839 182	1 111 110	592 592	N/A

WOI, window of implantation; Mean ± SD values for age and BMI of each group are shown; N/A, not applicable.

### Phenotypic analysis of immune cells

**Figure 2.**
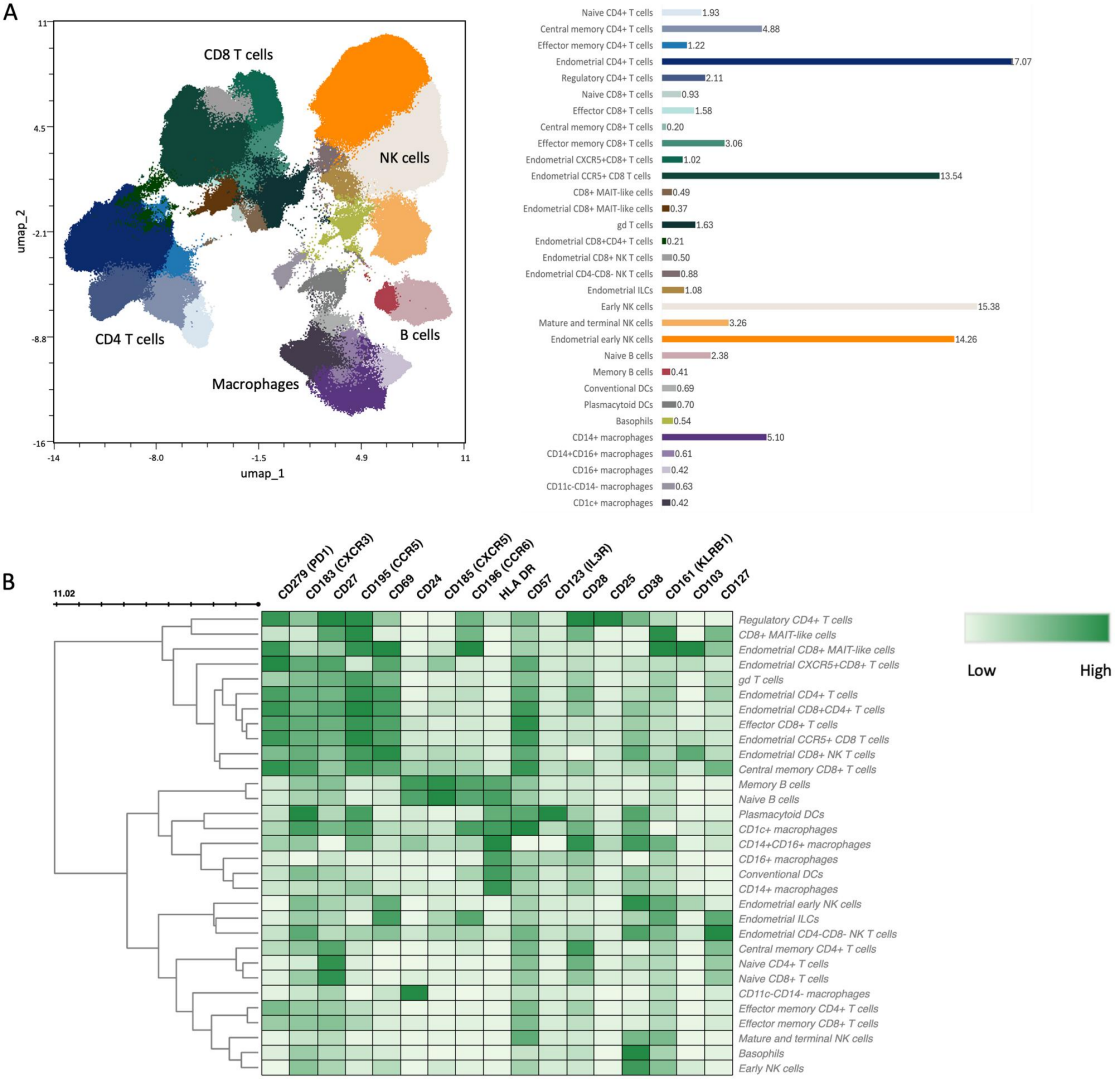
**Visualization of high-dimensional data analysis of endometrial immune cells.** (**A**) Single cell preparations of endometrial digests (endometriosis: n = 18 and controls: n = 12) taken from various points across the menstrual cycle were subjected to 36-parameter flow cytometry, and data acquired (Cytek Aurora). FlowSOM clustering of single/live endometrial CD45+ lymphocytes resulted in 31 immune cell clusters, which were mapped to two UMAP dimensions. Proportions of cells within the CD45+ population are shown (bars). (**B**) Heatmap of relevant marker expression across endometrial immune cell clusters, with hierarchical clustering showing relative levels of functional markers.

There were 883 181 endometrial CD45+ and 1 950 292 circulating CD45+ immune cells which passed through quality control checks and were included in our analysis (Table 3: All Participants). We identified 31 endometrial immune cell clusters as illustrated using UMAP (Fig. 2A) which were annotated based on the existing literature and a manual gating strategy (Park *et al.*, 2020). A heatmap of key protein expression data indicating immune cell function (e.g. PD-1, HLA-DR), lineage subtype (CD27, CD25, CD127, etc), tissue residency (CD103, CD69), and chemokine receptor expression (CXCR3, CCR5, CXCR5, etc), for the 31 clusters is shown in Fig. 2B. Based on different phenotypic traits and established markers of tissue residency, clusters expressing CD69 and CD103 were identified and characterized as ‘endometrial’. Overall, T cells were the main population (38.18%), followed by NK cells (28.96%) and macrophages (7.17%). Not accounting for unconventional subtypes, the CD4/CD8 ratio was slightly in favour of CD4 T cells, with 19.97% versus 18.20% of all endometrial leukocytes. The majority of CD8+ subtypes were tissue resident clusters, including endometrial CXCR5+ and CCR5+ CD8 T cells. Specific T cell subpopulations were also detected, including Treg (2.11%), γδ T cells (1.63%), and double positive (CD4+CD8+) T cells (0.21%). Also, minority populations were present, such as B cells (2.79%), dendritic cells (1.39%), and basophils (0.54%).

### Endometrial immune across the menstrual cycle

**Figure 3.**
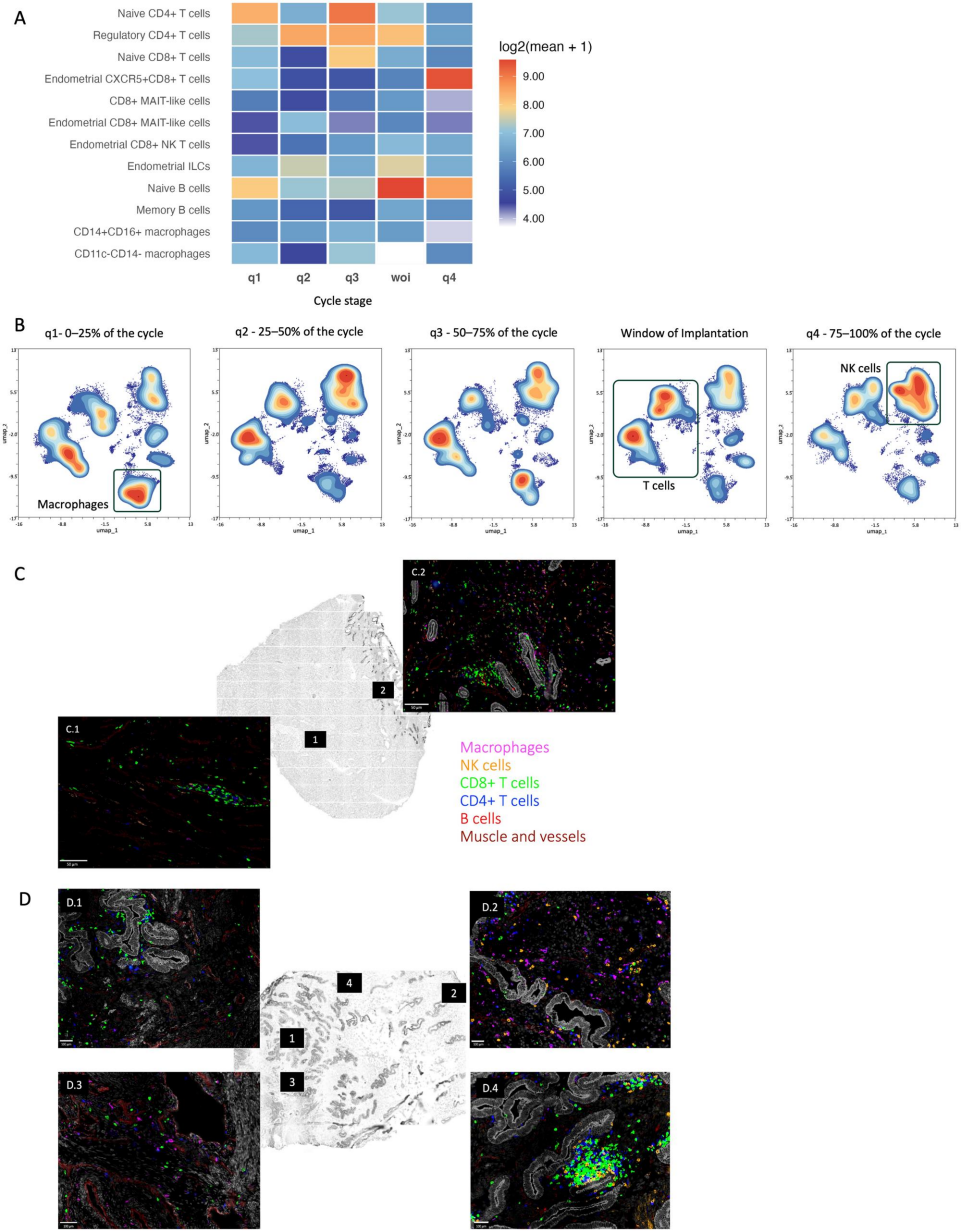
**Dynamic changes in endometrial immune cell populations across the menstrual cycle.** (**A**) Heatmap showing mean normalized counts of 12 significantly changed immune cell clusters summarized by menstrual cycle quarter (q1, q2, q3, window of implantation, q4). Colour and its intensity represent log2-transformed mean abundance [log2(mean + 1)], with blue indicating lower values and orange indicating higher values, while white denotes zero or near-zero abundance. Each row corresponds to a single immune cell cluster; values are descriptive summaries only. No statistical testing or uncertainty estimates are shown for this visualization. (**B**) Qualitative assessment of immune changes across the menstrual cycle, analysed with FlowSOM and visualized with UMAP via Omiq (Dotmatics, USA), showing the contribution of cells from each phase of the menstrual cycle. (**C, D**) Multiplex imaging of immune cells in endometrial and myometrial tissue sections, in first quarter of the menstrual cycle (C) and in the fourth quarter of the menstrual cycle (D). False-colour fluorescence imaging macrophages (pink – CD14, CD68), natural killer (NK) cells (orange – CD56), CD8+ T cells (green), CD4+ T cells (blue), B cells (red – CD19, CD20), epithelium (light grey – pan-cytokeratin), cell nuclei (dark grey – hoechst), muscle cells (dark red – SMA), and blood vessels (dark red – CD26, CD31). Multiplex imaging was performed with the ZellScannerONE™ ChipCytometry™ instrument. Specific highlighted regions are (C.1) myometrium section, (C.2) basal and functional endometrium, (D.1) myometrium: endometrium junction, (D.2) luminal epithelium, (D.3) myometrium, (D.4) basal endometrium.

We investigated the impact of menstrual cycle phase and patient characteristics on endometrial immune cells. The menstrual cycle phase was separated into four quarters, with additional consideration of WOI when known. Cell populations were compared to the preceding phase, revealing significant phase-dependent fluctuations across multiple immune cell clusters (n = 12), visualized in the cycle-quarter heatmap (Fig. 3A) and on UMAP projections of the total immune repertoire (Fig. 3B). Each of the 31 clusters (including non-significantly altered datasets) is displayed, comparing normalized cell counts versus cycle phase, separating endometriosis and control datapoints (Supplementary Fig. S4). In general, macrophages dominated in the early stages of the cycle. As the cycle advanced, there was a notable increase in T cells and NK cells. T cells were the most abundant population during WOI while in the final quarter of the cycle NK cells were the predominant cell type. Considering sub-populations, naive CD4+ and CD8+ T cells peak around the mid-cycle, naïve B cells peak during WOI, while CXCR5+CD8+ T cells surge near the end of the cycle, close to 100%. We visualized the presence of immune cells in the proliferative versus secretory phase of the menstrual cycle using chip cytometry for spatial distribution (Fig. 3C and D), focusing on markers of the most altered cell populations: macrophage, CD8 and CD4 T cells, B cells, and NK cells. In the early proliferative phase (comparable to the first quarter), CD8 T/NK/CD4 T/B cells and macrophage were found as single cells in the endometrium, with CD8 T cells often clustering close to glandular epithelium (Fig. 3C.2). These were all more abundant than in the deeper myometrium, where CD8 T cells appeared to predominate (Fig. 3C.1). In the late secretory phase (comparable to the fourth quarter), macrophage and NK cells were most abundant in the outer functionalis (Fig. 3D.2) whereas CD8 T cells were often found in clusters nearer the basalis (Fig. 3D.4) or nearer to luminal epithelium (Fig. 3D.1); NK and CD4 T were found throughout.

**Figure 4.**
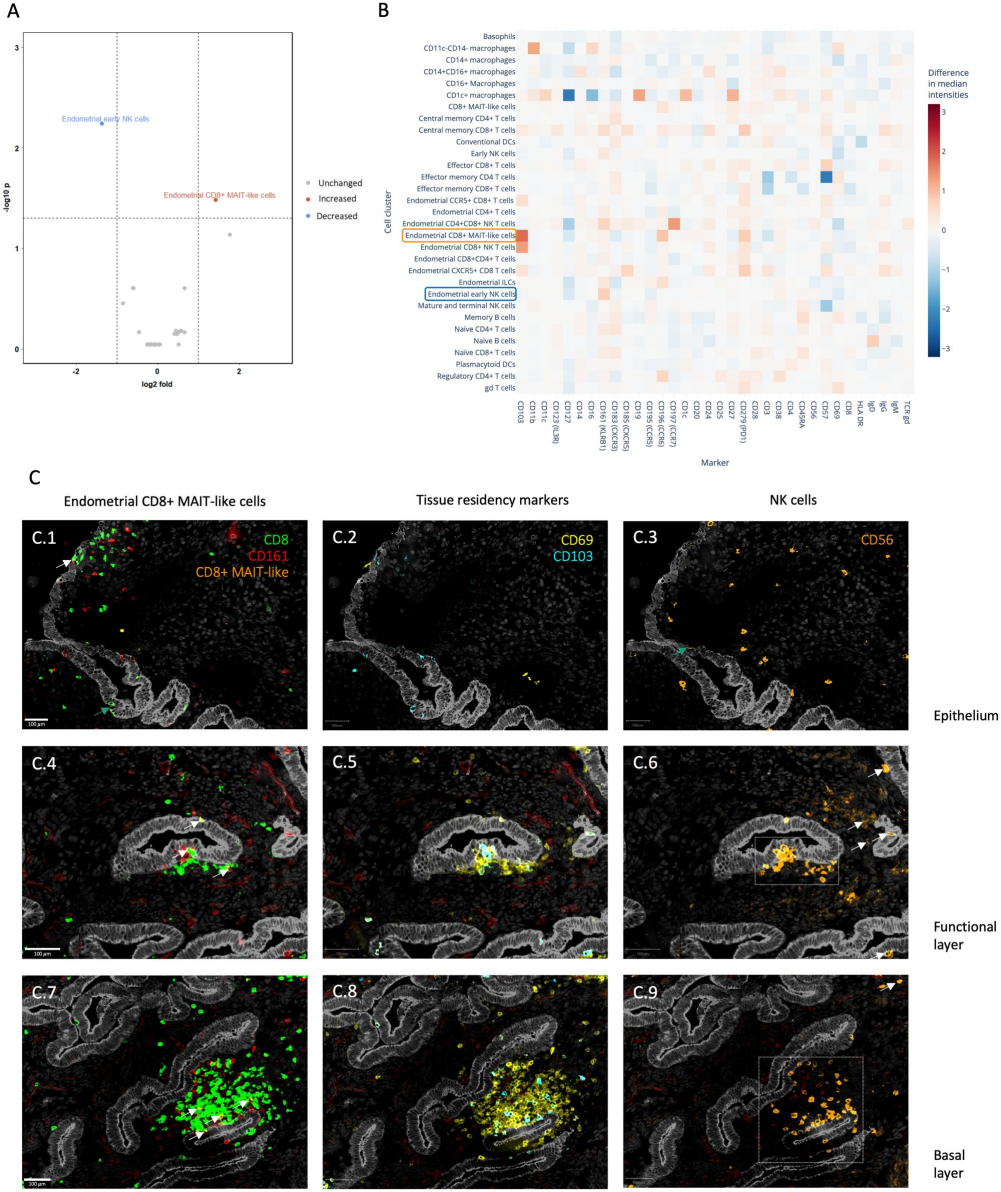
**Altered endometrial immune cell populations and marker expression between patients with endometriosis and non-endometriosis controls.** (**A**) Volcano plot shows significantly changed endometrial cell clusters in endometriosis patients compared to healthy controls. Each point represents one cell cluster. Adjusted *P*-values < 0.05 and |log2FC|>1 were applied. Analysis was performed on 861 796 cells from 25 patients with DESeq2 (Love *et al*., 2014) and visualized with OmiqM (Dotmatics, USA). (**B**) Heatmap depicting markers that are significantly different between endometriosis and control groups, based on differences in median expression intensity. Adjusted *P*-values < 0.05 are plotted as colours on a grid of the cell clusters on the vertical axis and the markers on the *x*-axis. The rejected adjusted *P*-values are omitted. Differences in median expression levels are displayed using a colour scale, with darker colours indicating bigger difference in expression between the two groups. Data were visualized using the Python library Seaborn. (**C**) Image shows a section of full-thickness endometrial biopsy taken from a fertile patient without endometriosis. Endometrial subtypes of CD8+ Mucosal-Associated Invariant T-like (MAIT-like) and Natural Killer (NK) cells, indicated with white arrows, were determined by colocalization of tissue residency markers CD69+ and CD103+ (2, 5, and 8) and respective lineage markers, CD8+ and CD161+ for MAIT-like and CD56+ for NK cells. Endometrial CD8+ MAIT-like cells in orange on the left and NK cells on the right were present as single cells in the luminal (1 and 3) and glandular epithelium (4 and 6), across the endometrial stroma and (6 and 9) in the lymphoid aggregates (7 and 9). False-colour fluorescence imaging for CD8+ T cells in green, CD161+ cells in red, CD8+ MAIT-like cells (CD8 and CD161) in orange left, CD69+ in yellow, CD103+ in blue, CD56+ in orange right, epithelium in grey (pan-cytokeratin) and cell nuclei in dark grey (hoechst). Multiplex imaging was performed with ZellScannerONE™ ChipCytometry™ instrument.

Next, we conducted differential analyses of endometrial CD45+ immune cell populations with respect to demographic variables (Table 3). Factors such as age under/over 35 years and BMI under/over 30 kg/m2 did not significantly influence any of the 31 endometrial immune cells (*P*.adj < 0.05, |log2FC| > 1, data not shown). However, a history of miscarriage and previous live birth was revealed to have a significant influence on immune cell populations. Decreased levels of CD8+ MAIT-like cells in the participants with a history of miscarriage were found in the endometrium (*P*.adj = 0.023, log2FC = −1.900), while effector CD8 T cells were increased in the same group in peripheral blood (*P*.adj = < 0.001, log2FC = 2.017). Parous participants had increased levels of CD8+ MAIT cells (*P*.adj = 0.004, log2FC = 2.190) and decreased levels of effector CD8 T cells (*P*.adj = 0.002, log2FC = −1.870) in the endometrium. Importantly in further analysis, these factors that impact endometrial immune cell composition were controlled for in all cytometric data analyses.

### The influence of endometriosis on endometrial immune cells

Differential analysis was performed on 861 796 cells from 25 patients, endometriosis (n = 17) and non-endometriosis control group (n = 8) (Table 3: Case: Control Cohort). The endometrial CD8+ MAIT-like cell cluster proportion was increased (*P*.adj = 0.033, log2FC = 1.415) while endometrial early NK cells were decreased (*P*.adj = 0.006, log2FC = −1.369) in the endometriosis group (Fig. 4A). No differences were observed between endometriosis patients with stage I/II versus III/IV disease (data not shown).

We further explored endometrial CD8+ MAIT-like cells, which represented 0.37 ± 0.51% of the total CD45+ population, and endometrial early NK cells, which represented 14.26 ± 9.53% of the total CD45+ population. In endometriosis patients, compared to controls, both endometrial CD8+ MAIT-like (blue box) and early NK cells (red box) displayed significantly higher levels of tissue-homing markers CD103 and CCR6 as well as regulatory PD-1 while having a lower expression of markers indicating cell differentiation and activation CD127, CD27, and CD69 (Fig. 4B). We also investigated the marker expression patterns in endometrial immune clusters between the groups and revealed distinct differences in various populations (Fig. 4B). The expression of CD161, CCR6, and PD-1 was significantly increased across most clusters in the endometriosis group. CD161 is a marker of all human IL-17-producing subsets; CCR6 is a chemokine receptor important for cell trafficking into mucosal lymphoid tissues; and PD-1 is a cell surface receptor protein that plays a crucial role in immune regulation by inhibiting T cell activation and promoting immune tolerance.

To complement the flow cytometry analysis findings, we visualized endometrial CD8+ MAIT-like cells and endometrial early NK cells in three full-thickness biopsy samples of the luminal edge, functional layer, and the basal endometrium (Fig. 4C). First, CD8+ MAIT-like cells were determined by co-expression of CD8 and CD161 (Fig. 4C.1, 4C.4, and 4C.7) and NK cells were determined by the expression of CD56 (Fig. 4C.3, 4C.6, and 4C.9). Both populations were found across the epithelium, functional, and basal layer of the endometrium, and in the epithelium as well across the stroma and in lymphoid aggregates. Next, tissue-resident populations were identified with CD103 and CD69 expression (Fig. 4C.2, 4C.5, and 4C.8), which tended to be higher in cells in the functionalis/basal endometrium rather than the luminal edge.

### MAIT cells in the endometrium of women with endometriosis

In the following validation study, 49 360 MAIT cells (effector memory CD161+Vα7.2+) were detected in the eutopic endometrium of eight endometrial samples from endometriosis patients (Table 3: Validation cohort, with participants distinct from prior cohorts) using a specifically designed MAIT panel (Table 2). They constituted 3.83% of total T cells and peaked during ovulation and the WOI compared to earlier menstrual phases (*P*.adj < 0.05). Comparison of the deep immunophenotyping and validation panels demonstrated good correspondence in the tissue-resident compartment: in the deep immunophenotyping panel, endometrial MAIT-like cells (CD161+CD69+CD103+) averaged 1.38%, while in the validation panel, endometrial MAITs (CD161+TCRVα7.2+CD69+CD103+) averaged 2.38% of T cells. For non-resident cells, the deep immunophenotyping panel reported 2.09% MAIT-like cells (CD161+CD69-CD103-), whereas the validation panel identified 1.45% CD69-CD103-MAITs. Notably, the MAIT-like populations defined in the deep immunophenotyping panel correlated more closely to true MAITs (CD161+TCRVα7.2+) than CD161+ cells lacking TCRVα7.2 (13.72% CD161+TCRVα7.2-CD69+CD103+; 0.05% CD161+TCRVα7.2-CD69-CD103-), supporting the accuracy of clustering in the case: control dataset from the main study and highlighting MAITs as a population of interest in endometriosis.

### Peripheral blood immune cells in endometriosis

**Figure 5.**
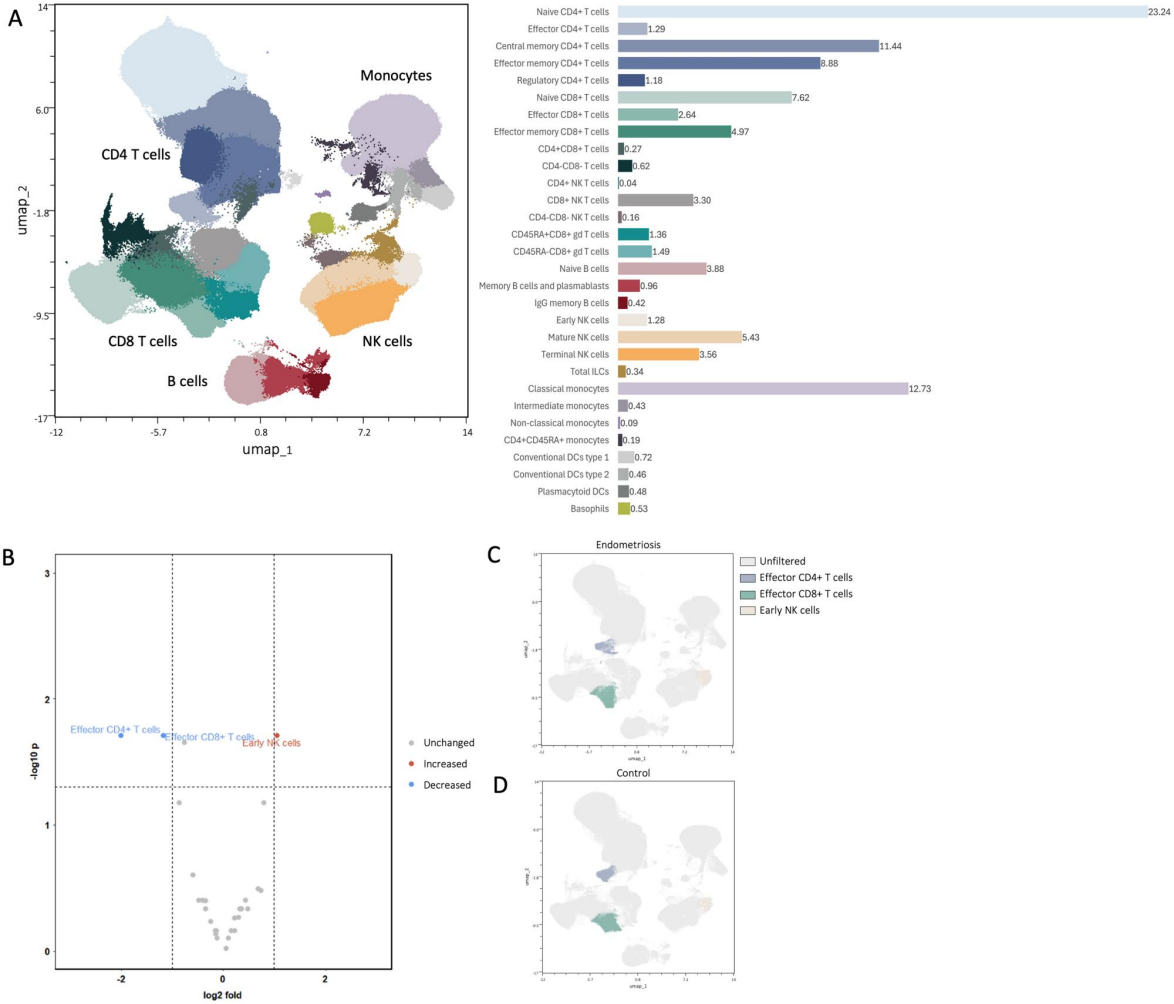
**Altered circulating immune cell populations between patients with endometriosis and non-endometriosis controls.** (**A**) FlowSOM clustering was performed on single circulating CD45+ leukocytes and resulted in 30 immune cell clusters, which were mapped to the two UMAP dimensions. Altogether, 1 950 292 cells from 27 participants were analysed. Data were analysed with FlowSOM and visualized with UMAP via OmiqM (Dotmatics, USA). (**B**) Volcano plot shows statistically significantly changed circulating cell clusters in endometriosis patients compared to healthy controls. Each point represents one cell cluster, which was coloured according to the figure legend. *P*.adj < 0.05 and |log2FC|>1 were applied. Analysis was performed on 1 703 702 cells from 23 patients with DESeq2 (Love *et al*., 2014) and visualized with Omiq (Dotmatics, USA). (**C**) High-dimensional data visualization of circulating immune populations from the endometriosis group displaying altered cell clusters projected onto two UMAP dimensions. (**D**) High-dimensional data visualization of circulating immune populations from the control group displaying altered cell clusters projected onto two UMAP dimensions. In parts C and D, 592 591 cells from each patient group were mapped to the two UMAP dimensions; each dot represents one cell.

Analysis of peripheral blood immune cells using unsupervised clustering (Table 3: All participants) delineated 30 immune cell clusters from 1 950 292 cells from 27 participants (Table 3, Fig. 5A). Unsurprisingly, few (<2% of CD45+ cells) tissue resident (CD103/CD69) clusters were identified as they are minor populations in the blood. In contrast to the endometrium, where CD8 T cells predominate, CD4 T cells are the major immune cell population (43.92%), followed by monocytes (17.45%) and CD8 T cells (15.93%). Naïve T cells are the majority population (for both CD4 and CD8 positive cells) in the blood, but few exist in the endometrium.

We compared cell population phenotypes and cell counts between endometriosis and control patients (Table 3: Case: Control cohort) and observed systemic changes in immune populations. The proportions of cell counts through each phase of the menstrual cycle (from endometriosis and control patients) can be seen in Supplementary Fig. S5. Significant changes were found across the cycle for seven populations: effector CD4 T, effector memory CD8 T, CD4-/CD8-T, CD4-/CD8-NKT, Terminal NK, classical monocytes, and CD4+/CD45RA+ monocytes (Supplementary Fig. S6). Differential analysis on these 1 703 702 circulating CD45+ cells from 23 participants revealed increased early NK cells (*P*.adj = 0.001 and log2FC = 1.052) and decreased effector CD4 T (*P*.adj = 0.002 and log2FC = −2.010) and effector CD8 T cells (*P*.adj = 0.002 and log2FC = −1.180) in the endometriosis group (Fig. 5B).

## Discussion

We describe the first phenotypic study on endometrial and matched circulating immune cells utilizing full-spectrum flow cytometry designed to profile a broad immune cell repertoire encompassing functional information. Analysing 883 181 endometrial and 1 950 292 circulating CD45+ cells across the menstrual cycle, this is the most extensive study to date investigating and comparing endometrial and systemic immunity. As expected, endometrial and peripheral blood immune populations varied in proportion and were phenotypically different, therefore, we conclude peripheral blood is a poor indicator of endometrial immune activity and should be mainly considered independently for diagnostic purposes. Endometrial immune cell analysis indicated that both a history of miscarriage and previous live birth influenced endometrial immune populations, whereas BMI and age did not. This is particularly important considering the recently implicated clinical overlap of miscarriage and endometriosis, which was also observed in our cohort (Boje *et al.*, 2023). This data indicates future clinical studies should be designed considering gravidity and parity. This is in agreement with the observation of pregnancy influence on NK cell populations (Gamliel *et al.*, 2018).

Our data indicated that the menstrual cycle phase influences endometrial immune cell populations, as previously reviewed (Wira *et al.*, 2015). NK cells are widely accepted to be increased above T cell populations in the secretory phase facilitating implantation potential; this is seen in our data (Fig. 3B). Our study permits immune lineage subpopulation analysis rather than broadly defining marker analysis for bulk populations, as has often been the case in prior conventional flow cytometry and immunohistochemistry studies. Considering cells from all samples across the menstrual cycle, we found slightly more total T cells than NK cells, and also the presence of macrophage, B cells, dendritic cells, MAIT-like cells, and basophils (in decreasing order of total prevalence). Our flow cytometry panel favoured phenotyping of T cell lineage, and we were able to further categorize naïve/memory/tissue resident cells and subpopulations such as double positive T cells, gamma delta-T, Treg, and MAIT-like cells. Comparing subsets across the menstrual cycle showed that while NK cell proportional enrichment is clear in the later phases of the menstrual cycle and macrophage are abundant just after menstruation in the early phases (Fig. 3B), subpopulations are more subject to fluctuations (Fig. 3A). Interestingly, given their known role in pregnancy tolerance (Murata *et al.*, 2021), Treg increase through the cycle with a decline in proportion after WOI. Naïve CD4 and CD8 T cells are increased in the midcycle, and naïve B cells increase in the WOI, possibly indicating recruitment from the peripheral blood at these time points. Endometrial CD8 T cells expressing CXCR5 were highly enriched in the later phase of the cycle preceding menstruation; these cells also highly express PD-1, CD69, and CXCR3 but lack CCR5 (Fig. 2B). CXCR5+ CD8 T cells are known for their role in controlling antibody production as this population is typically considered to be a follicular homing receptor, however in this endometrial context it is more likely that the cells are part of ‘lymphoid aggregates’ which are analogous to tertiary lymphoid structures (TLS) found in other human tissues (Gago da Graca *et al.*, 2021). Lymphoid aggregates are known to expand prior to menstruation (Wira *et al.*, 2015), we identified CD8 T cells clustering in the basal endometrium (Figs 3D.4 and 4C.7–9), and we have previously found the presence of CD4+ CXCR5+ Tfh-like cells in the endometrium. This enrichment of CXCR5+ CD8T cells could be an indication of TLS generation prior to menstruation, and merits further investigation.

Tissue resident CD8+ MAIT-like cells were found to be elevated in the endometrium of women with endometriosis. These cells are defined as ‘MAIT-like’ as our panel did not incorporate specificity for semi-invariant TCR Vα7.2-Jα33/12/20 TCR or MR1 binding capacity, therefore further work was required to validate this observation. In a separate validation panel, we identified the presence of TCR Vα7.2 positive CD161+CD8+T cells in the endometrium, which were at a very similar proportion to CD8+ MAIT-like (CD161+CD8+CD3+) cells identified in the primary case: control cohort. Imaging via multiplex chip cytometry revealed CD8+ MAIT-like cells were distributed as single cells scattered near and in the glandular epithelium but also among other CD8 T cells. In addition, all of the identified CD8+ MAIT-like (CD161+CD8+CD3+) cells also expressed CD69 and CD103 markers (Fig. 4C), confirming flow cytometry data that the majority of CD8+ MAIT-like cells are a tissue resident population.

MAIT cells have been implicated in the outcome of various diseases, from microbial infection and autoimmune inflammatory diseases to malignancy (Provine and Klenerman, 2020). MAIT cell frequency increases in infected and inflamed tissues through migration from peripheral blood or local proliferation at infected sites (Nel *et al.*, 2021). In endometriosis, CD4+ and CD8+ MAIT subtypes are known to be enriched in peripheral blood and peritoneal fluids compared to controls, combined with heightened MAIT-cell activation and IL-8, IL-12, and IL-17 production, control participants had increased double-negative subtypes and higher expression of PD-1 (Li *et al.*, 2019). CD161 is a key marker of MAIT cells, and is linked to specific cytokine production potential from T cells, such as IL-17. Our data show that in endometriosis, CD161 expression is heightened across all populations of cells. In the gut, locally increased expression of IL-17 by MAIT cells can be harmful for epithelial barrier integrity, thus aggravating infection and potentially facilitating dissemination of cancer metastases by inhibiting NK cell antimetastatic response and increasing blood vessel permeability (Kulig *et al.*, 2016; Lu *et al.*, 2020). Dysregulation in the IL-17 axis is linked to subfertility, therefore the dysregulation identified here may contribute endometriosis-associated subfertility. This is particularly interesting in the context of the lymphatic and vascular metastasis hypothesis of the endometriosis histogenesis (Mechsner *et al.*, 2008; Zondervan *et al.*, 2018; Samani *et al.*, 2019). Findings that could support this include increased levels of IL-18, an important mediator of TCR-independent MAIT cell activation, in the peritoneal fluid of patients with endometriosis, and increased levels of IL-17 found in stage I/II disease compared to stage III/IV and control participants (Oku *et al.*, 2004; Zhang *et al.*, 2005; Ayaz *et al.*, 2011).

Uterine NK cells are the predominant immune cell in early pregnancy decidua, due to their important role in embryo implantation and placentation, and disruptions are reported in various pregnancy pathologies (Shi *et al.*, 2025). Various changes in the uterine NK cell population have been identified in endometriosis: NKp46 is decreased in severe endometriosis and NKp30 is upregulated (Shi *et al.*, 2025); however our study, which favoured T cell lineage markers over NK cells, did not incorporate these markers, therefore we cannot replicate this observation. We found decreased early endometrial NK cells in endometriosis; further research into detailed NK cell phenotypic changes is warranted with an alternate targeted methodology.

Despite the limitations in inferring endometrial immune pathophysiology from the peripheral blood immune cells, systemic changes hold great diagnostic potential. Systemic immunity in patients with endometriosis was analysed, while carefully controlling for the menstrual cycle phase, parity, and miscarriage. We identified that effector CD4 and CD8 T cells were reduced, and early NK cells increased in the peripheral blood of women with endometriosis. In addition to already established associations with some autoimmune diseases (Shigesi *et al.*, 2025), systemic immune changes in endometriosis were also suggested in the recent GWAS study on common genetic variants (Shigesi *et al.*, 2019; Rahmioglu *et al.*, 2023). Significant changes in gene expression or methylation were revealed, among others, for genes ESR1 and SYNE1, implicated in CD8 T cell function modulation via oestrogen and TCR signalling, respectively (Li *et al.*, 2020; Yuan *et al.*, 2021). Given the invasiveness of endometrial sampling, this could significantly aid the development of non-invasive diagnostics as no reliable diagnostic biomarker exists (Dolińska *et al.*, 2023).

### Limitations to the study

In interpreting our findings, several methodological considerations are relevant. Although histological cycle dating was not available, modelling menstrual phase using cycle percentages and predefined quarters in participants with regular cycles enabled high-resolution comparison of immune variation across the menstrual cycle. The use of high-dimensional single-cell profiling increased sensitivity to studying endometrial and circulating immune populations that may be overlooked by conventional mean-based approaches. However, the cross-sectional design and stringent eligibility criteria limited sample size and precluded inference of temporal or causal relationships, as well as more granular stratification by clinical phenotype, including endometriosis-associated subfertility, which is of particular interest in the context of eutopic endometrial immunity. In addition, the marked divergence observed between peripheral and endometrial immune compartments indicates that systemic and local immune findings should be interpreted within their specific biological context, particularly when considering diagnostic utility versus implications for endometrial pathophysiology and lesion biology. Importantly, we did not study the immunological niche of endometriotic lesions, and our data therefore have limited ability to inform lesion-directed immunotherapies.

## Conclusions

This study advances the translational endometriosis literature by resolving immune phenotypes at single-cell level across menstrual cycle and anatomical compartments, providing a framework to guide both target selection and sampling strategy in future work. The distinct immune signatures observed in peripheral blood and eutopic endometrium emphasize that circulating changes are most relevant to the development of non-invasive diagnostics and patient stratification, whereas endometrial alterations are more plausibly linked to receptivity, fertility, and implantation. In this context, the increase in tissue-resident CD8+ MAIT-like cells together with reduced endometrial early NK cells highlights immune pathways with established relevance to mucosal tolerance, IL-17-inflammation, and uterine immune support of implantation, providing a rationale for mechanistic studies focused on endometriosis-associated subfertility. Our findings further underscore the importance of accounting for menstrual cycle timing and reproductive history when designing and interpreting immunological studies in endometriosis, as both exert substantial influence on endometrial immune composition. The populations highlighted here should be viewed as candidates for focused studies on eutopic endometrial dysfunction and adverse reproductive outcomes. This compartment-aware approach will be essential to maximize reproducibility and translational impact, and to avoid conflating systemic biomarkers with local pathophysiology.

## Supplementary Material

deag090_Supplementary_Materials_and_Methods

deag090_Supplementary_Figure_S1

deag090_Supplementary_Figure_S2

deag090_Supplementary_Figure_S3

deag090_Supplementary_Figure_S4

deag090_Supplementary_Figure_S5

deag090_Supplementary_Figure_S6

deag090_Supplementary_Table_S1

deag090_Supplementary_Table_S2

deag090_Supplementary_Table_S3

deag090_Supplementary_Table_S4

## Data Availability

The datasets generated and analysed during the current study are not publicly available, but are available from the corresponding author upon reasonable request. Analysis code used for data processing and statistical analyses is also available upon reasonable request.
