# Supplementary material for: Deep immune profiling of endometrial and peripheral blood cells in endometriosis
Source: Hum Reprod. 2026 Jun 5;41(8):1324–37. doi: 10.1093/humrep/deag090 (PMC13429876; doi:10.1093/humrep/deag090)
Supplement: deag090_Supplementary_Materials_and_Methods [file deag090_supplementary_materials_and_methods.pdf]

Human Reproduction, 2026, 1–1
https://doi.org/10.1093/humrep/deag090
Supplementary Data

human reproduction
OXFORD

Full-spectrum flow cytometry study protocol

Sample processing protocol

Materials and reagents

Cryovials
Fetal Calf Serum (FCS) (Sera Laboratories International Ltd, EU-000-F)
PBS (SIGMA, D8537)
LymphoprepTM (Axis Shield)
Iscove's Modified Dulbecco's Medium (Sigma, 13390)
DNase (Sigma, 10104159001)
DMSO (Sigma, 5.89569)

Reagent's preparation

- Digestion Buffer: Add 50 ml FCS, 5 ml Human Serum, and 5 ml p/s/g (100 IU/ml penicillin, 100 g/ml streptomycin, and 2 mM glutamine) into 500 ml Iscove's Modified Dulbecco's Medium.
- DNase I aliquot: DNase I powder in 0.15 M NaCl (5 mg/ml), 10 mg/ml 100 µl stock aliquots
- Freezing Media (10% DMSO): 10 ml DMSO + 90 ml FCS

Isolation of peripheral blood mononuclear cells (PBMCs)

1. Prepare PBS/2% FCS, at room temperature (15–25 °C).
2. Transfer 4 ml blood from Lithium/Sodium Heparin vacutainer to a 15 ml tube, dilute with 4 ml of PBS (equal volume).
3. Add 3 ml Lymphoprep™ in a separate 15 ml tube.
4. Gently lay 8 ml of diluted blood on top of Lymphoprep™ in each 15 ml tube.
5. Centrifuge at 800 *g*, room temperature for 20 min without centrifugal brake.
6. After centrifugation, carefully remove the PBMCs layer in the Lymphoprep™/plasma interface with a transfer pipette into a 15 ml tube.
7. Top up the 15 ml tube to 15 ml with PBS/2% FCS. Centrifuge for 10 min at 300 *g*, room temperature. Aspirate the supernatant. Repeat.
8. Re-suspend cell pellet in 1 ml PBS.
9. Transfer 10 µl of collected cell suspension to 1.5 ml Eppendorf. Keep the 15 ml tube with cell suspension on ice.
10. Dilute cell sample in Trypan Blue by preparing a 1:1 dilution of the cell suspension (10 µl + 10 µl) using a 0.4% Trypan Blue solution.
11. Perform cell counting using a Neubauer counting chamber.

Tissue digestion and isolation of HECs

1. Ensure sterility. Work in a Class II microbiological safety cabinet and ensure full aseptic technique.
2. Prepare Digestion Buffer and warm to 37 °C. Turn on the shaking incubator and set the temperature to 37 °C.
3. Transfer endometrial tissue with PBS to a Petri-dish. Record shape, size, and patient number of the biopsy. Take a photo of the sample.
4. Take 10 mm^3 of the tissue, cut into 1 mm^3 fragments using scalpel and pipette, and remove mucus. Ensure tissue fragments are small enough to fit through the tip of the transfer pipette (excess ES with good structure will be snap-frozen for immunohistochemistry).
5. Transfer all the tissue fragments into 15 ml tubes, maximum 20 mm^3 per tube (I usually do around 500ul amount of tissue in each tube–that is the tissue size reach 500 µl in 15 ml tube). If blood contamination exchange solution to PBS. Remove supernatant to 1 ml solution of PBS with endometrial tissue.
6. Add 10 ml Digestion Buffer to each 15 ml tubes. Remove the excess buffer with only 1 ml left in each tube.
7. Add 100 µl DNase I aliquot to each 15 ml tube, vortex for 20 s.
8. Add 10 ml Digestion Buffer to each 15 ml tubes and pass through sequential 70 and 40 µm cell strainers, wash the cell strainers twice with 3 ml Digestion Buffer, collect all the run-through in a new 50 ml Falcon tube.
9. Centrifuge the collected run-through for 10 min at 300 *g*, room temperature, aspirate the supernatant then re-suspend cell pellet in 1 ml PBS.
10. Transfer 10 µl of collected cell suspension to 1.5 ml Eppendorf. Keep the 15 ml tube with cell suspension on ice.
11. Dilute cell sample in Trypan Blue by preparing a 1:1 dilution of the cell suspension using a 0.4% Trypan Blue solution.
12. Carefully and continuously fill the haemocytometer chamber.
13. Incubate the haemocytometer and cells for 1–2 min at room temperature.
14. Perform cell counting.

Cell storage

- Transfer the rest of cells to one Eppendorf, centrifuge for 5 min at 300 *g*. Aspirate all supernatant then re-suspend cell pellet with 1 ml Freezing Media.
- Transfer 1 ml cell suspension to one labelled Cryo tube vial (sample number, cell type, cell number, date, name, number of samples), place the vial in Cryo Freezing container with isopropyl alcohol, stored in −80°C overnight.

Staining protocol

Materials and reagents

RPMI 1640-Medium (Sigma, R8758)
Fetal Calf Serum (FCS) (Sera Laboratories International Ltd, EU-000-F)
PBS (Wash buffer) (SIGMA, D8537)
Zombie NIR™ dye (Biolegend, 423106)
DMSO (Sigma, 5.89569)
Cell Staining Buffer (Stain buffer) (Biolegend, 420201)
Brilliant Stain Buffer Plus (BD Biosciences, 563794)
True-Stain Monocyte Blocker™ (Biolegend, 426103)
Specific antibodies (**Error! Reference source not found.**)
FluoroFix™ Buffer (Biolegend, 422101)

Reagent's preparation

1. Complete RPMI: Add 50 ml of FBS, 5 ml of Penicillin-Streptomycin into 500 ml of RPMI 1640 (with L-glutamine and sodium bicarbonate).

2. Zombie: Pre-warm the kit to room temperature; spin down the vial of lyophilized reagent in a microcentrifuge to ensure the reagent is at the bottom of the vial and add 100 µl of DMSO to one vial of Zombie NIR™ dye and mix until fully dissolved. Aliquot to 1 µl in an Eppendorf tube and store at −4°C.
3. Spin antibodies at 13 000 *g* for 1 s.

Thawing

1. Pre-warm 30 ml of complete RPMI at 37 °C for at least 30 min.
2. Prepare MULTI-ANTIBODY MIX excluding the antibodies listed below.
3. Thaw cryo-vial in 37 °C water bath, until only small piece of ice remains.
4. Transfer 10 ml of warm complete RPMI to 15 ml conical tube.
5. Add contents of cryo-vial with a transfer pipette.
6. Add additional volume of complete RPMI to complete to 15 ml.
7. Spin at 400 *g* for 10 min (1500 rpm for 10 min in the TC room).
8. Decant supernatant and resuspend pellet in 1 ml of warm complete RPMI.
9. If not ready to proceed:
10. Add complete RPMI to 15 ml.
11. Repeat steps 6 and 7.
12. Resuspend in 7.5 ml of complete RPMI or to an approximate concentration of 3.5×10^6/ml.
13. Leave in incubator until ready to proceed (rest is not necessary, but once staining has begun, samples will need to be processed without delay).

Single antibody control staining

1. Label Eppendort Tubes® for single antibody controls (SAC): *unstained*, *SAC-marker*
2. Add approximately 3×10^5 cells or beads to the unstained and each of the SAC tubes.
3. Add 1.5 ml PBS to SAC-Zombie NIR (ZNIR) tube.
4. Add 1.5 ml wash buffer to all other SAC controls.
5. Spin at 13 000 *g* for 1 s.
6. Decant supernatant and leave on ice.
7. Prepare antibody dilutions to the total volume of 140 µl and add to the SAC tubes.
8. Incubate on ice in the dark for 30 min.
9. After incubation, add 1 ml wash buffer to the SAC-ZNIR and MAC tubes.
10. Spin at 13 000 *g* for 1 s.
11. Decant supernatant and proceed with fixation.
12. If not ready to proceed:
13. Resuspend cell pellet in 1.5 ml BioLegend's Cell Staining Buffer.
14. Keep on ice in dark until ready to use.

Viability dye staining

1. Thaw 1 µl aliquot of Zombie NIR™ viability dye and dilute in 1.5 ml PBS.
2. Decant supernatant and resuspend cells in the SAC-ZNIR and MAC tube in diluted 100 µl Zombie NIR™ solution.
3. Vortex and incubate at room temperature in the dark for 15 min.
4. After incubation, add 1 ml wash buffer to the SAC-ZNIR and MAC tubes.
5. Spin at 13 000 *g* for 1 s.
6. Decant supernatant.
7. If not ready to proceed:
8. Resuspend cell pellet in 1.5 ml BioLegend's Cell Staining Buffer.
9. Keep on ice in dark until ready to use.

Multi-antibody cocktail staining

Label Eppendorf Tubes® for multiple antibodies cocktail (MAC) for samples:

Unstained
MAC-PBMC-Sample ID
MAC-HEC-Sample ID tubes

1. Add approximately 2×10^6 cells to each MAC tube with a transfer pipette.
2. Add 1 ml PBS to all tubes.
3. Spin at 13 000 *g* for 1 s.
4. Add 10 µl of Brilliant Stain Buffer Plus to all MAC tubes, vortex well.
5. Add 5 µl of True-Stain Monocyte Blocker, vortex.
6. CHEMOKINE RECEPTORS FIRST
7. Add anti-CXCR5 (1.2 µl), vortex.
8. Add anti-CCR6 (1.25 µl), vortex.
9. Add anti-CCR7 (5 µl), vortex.
10. Add anti-CCR5 (2.5 µl), vortex.
11. Incubate for 10 min at room temperature in the dark.
12. Add anti-TCR$\gamma\delta$ (1.2 µl), vortex.
13. Add anti-IgD, (0.6 µl), vortex.
14. Add anti-PD-1 (5 µl), vortex.
15. Incubate for 10 min on ice in the dark.
16. Add anti-CD20 (5 µl), vortex.
17. Add anti-CD161 (5 µl), vortex.
18. Add anti-CD28 (2.5 µl), vortex.
19. Incubate for 10 min on ice in the dark.
20. Add the remainder of antibodies from the MAC, vortex.
21. Incubate for 30 min on ice in the dark.
22. After incubation, add 1 ml wash buffer to the SAC-ZNIR and MAC tubes.
23. Spin at 13 000 *g* for 1 s
24. Decant supernatant and proceed with fixation.
25. If not ready to proceed:
26. Resuspend cell pellet in 1.5 ml BioLegend's Cell Staining Buffer.
27. Keep on ice in dark until ready to use.

Fixation

1. Resuspend pellet in 250 µl of FluoroFix™ Buffer including SAC tubes, vortex.
2. Incubate for 15 min at room temperature.
3. Add 1 ml of wash buffer.
4. Spin at 13 000 *g* for 1 s.
5. Resuspend pellet in 250 µl of wash buffer.
6. Store at 4 °C protected from light until ready to acquire on the instrument.
