# Supplementary material for: Deep immune profiling of endometrial and peripheral blood cells in endometriosis
Source: Hum Reprod. 2026 Jun 5;41(8):1324–37. doi: 10.1093/humrep/deag090 (PMC13429876; doi:10.1093/humrep/deag090)
Supplement: deag090_Supplementary_Figure_S1 [file deag090_supplementary_figure_s1.pdf]

Human Reproduction, 2026, 1–1
https://doi.org/10.1093/humrep/deag090
Supplementary Data

human reproduction

OXFORD

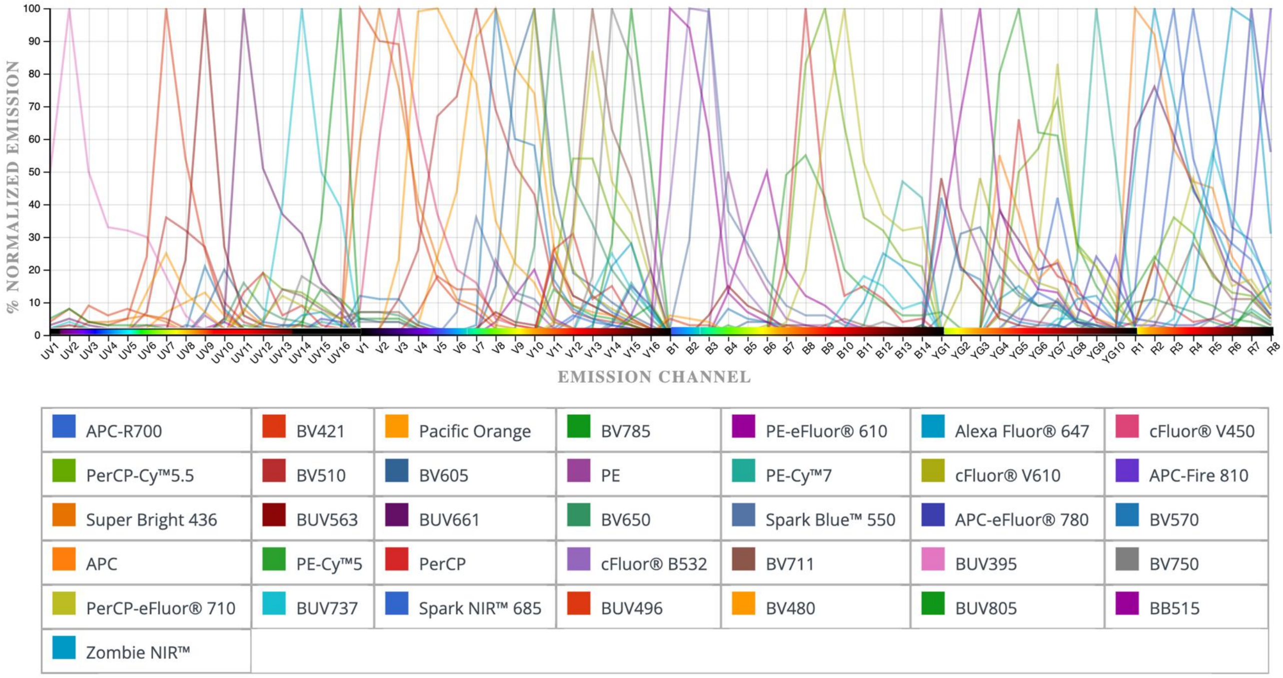

Supplementary Figure S1. Spectral signatures of 36 fluorochromes included in the panel. The Cytek Spectrum Viewer was used to visualize the spectral fingerprints of 36 fluorochromes. For direct comparison, all signatures were normalized to peak channels.
