# Supplementary material for: Deep immune profiling of endometrial and peripheral blood cells in endometriosis
Source: Hum Reprod. 2026 Jun 5;41(8):1324–37. doi: 10.1093/humrep/deag090 (PMC13429876; doi:10.1093/humrep/deag090)
Supplement: deag090_Supplementary_Figure_S2 [file deag090_supplementary_figure_s2.pdf]

Human Reproduction, 2026, 1–1
https://doi.org/10.1093/humrep/deag090
Supplementary Data

human reproduction OXFORD

Similarity™ Indices

Configuration:5L 16UV-16V-14B-10YG-8R

	APC-R700	BV421	Pacific Orange	BV785	PE-eFluor 610	Alexa Fluor 647	cFluor V450	PerCP-Cy5.5	BV510	BV605	PE	PE-Cy7	cFluor V610	APC-Fire 810	Super Bright 436	BUV563	BUV661	BV650	Spark Blue 550	APC-eFluor 780	BV570	APC	PE-Cy5	PerCP	cFluor B532	BV711	BUV395	BV750	PerCP-eFluor 710	BUV737	Spark NIR 685	BUV496	BV480	BUV805	BB515	Zombie NIR
APC-R700	1	0	0.01	0.07	0.04	0.63	0	0.41	0.01	0.05	0.01	0.1	0.07	0.2	0	0.01	0.5	0.18	0	0.32	0.02	0.57	0.28	0.17	0	0.39	0	0.11	0.49	0.45	0.78	0	0	0.04	0	0.51
BV421	0	1	0.07	0.07	0	0	0.78	0	0.17	0.06	0	0	0.01	0	0.97	0.01	0	0.09	0.02	0	0.15	0	0	0.01	0	0.09	0.05	0.05	0	0	0	0.09	0.28	0.01	0	0
Pacific Orange	0.01	0.07	1	0.03	0.11	0	0.17	0.04	0.88	0.62	0.16	0	0.46	0	0.09	0.14	0.04	0.25	0.33	0	0.77	0.03	0.02	0.03	0	0.08	0.01	0.05	0.04	0.02	0.01	0.22	0.58	0	0	0.01
BV785	0.07	0.07	0.03	1	0	0.01	0.06	0.19	0.03	0.07	0	0.17	0.06	0.18	0.07	0	0.05	0.15	0.01	0.22	0.03	0.04	0.03	0.07	0	0.49	0	0.82	0.26	0.25	0.04	0	0.02	0.21	0	0.27
PE-eFluor 610	0.04	0	0.11	0	1	0.05	0	0.18	0.07	0.47	0.59	0.04	0.54	0.01	0	0.32	0.11	0.14	0.16	0.01	0.35	0.14	0.36	0.21	0.05	0.03	0.01	0.01	0.11	0.01	0.03	0.01	0.03	0	0.03	0.01
Alexa Fluor 647	0.63	0	0	0.01	0.05	1	0	0.29	0	0.03	0.01	0.03	0.06	0.12	0	0.01	0.71	0.18	0	0.2	0.01	0.9	0.32	0.23	0	0.18	0	0.02	0.23	0.22	0.92	0	0	0.02	0	0.27
cFluor V450	0	0.78	0.17	0.06	0	0	1	0.01	0.36	0.07	0.01	0	0.02	0	0.86	0.01	0	0.08	0.05	0	0.15	0	0	0.01	0	0.07	0.01	0.04	0	0	0	0.13	0.58	0	0	0
PerCP-Cy5.5	0.41	0	0.04	0.19	0.18	0.29	0.01	1	0.02	0.17	0.05	0.23	0.22	0.09	0.01	0.03	0.39	0.38	0.06	0.15	0.06	0.37	0.72	0.81	0	0.5	0	0.28	0.86	0.31	0.35	0.01	0.01	0.06	0.01	0.25
BV510	0.01	0.17	0.88	0.03	0.07	0	0.36	0.02	1	0.4	0.12	0	0.27	0	0.22	0.22	0.04	0.16	0.3	0	0.54	0.02	0.01	0.01	0.02	0.06	0.02	0.04	0.02	0.02	0	0.48	0.84	0	0.01	0.01
BV605	0.05	0.06	0.62	0.07	0.47	0.03	0.07	0.17	0.4	1	0.28	0.03	0.89	0.01	0.06	0.2	0.17	0.53	0.16	0.02	0.7	0.14	0.21	0.18	0.01	0.17	0.01	0.1	0.14	0.05	0.03	0.05	0.16	0.01	0	0.02
PE	0.01	0	0.16	0	0.59	0.01	0.01	0.05	0.12	0.28	1	0.02	0.22	0	0.01	0.51	0.03	0.05	0.24	0	0.52	0.04	0.14	0.06	0.09	0.01	0.01	0	0.03	0	0.01	0.03	0.05	0	0.05	0
PE-Cy7	0.1	0	0	0.17	0.04	0.03	0	0.23	0	0.03	0.02	1	0.04	0.2	0	0.01	0.05	0.04	0.01	0.3	0.01	0.05	0.14	0.09	0	0.11	0	0.15	0.29	0.14	0.04	0	0	0.08	0	0.26
cFluor V610	0.07	0.01	0.46	0.06	0.54	0.06	0.02	0.22	0.27	0.89	0.22	0.04	1	0.02	0.01	0.12	0.17	0.56	0.11	0.02	0.53	0.19	0.31	0.23	0	0.17	0.01	0.09	0.16	0.04	0.06	0.02	0.1	0	0	0.02
APC-Fire 810	0.2	0	0	0.18	0.01	0.12	0	0.09	0	0.01	0	0.2	0.02	1	0	0	0.1	0.05	0	0.75	0	0.13	0.06	0.03	0	0.12	0	0.12	0.13	0.21	0.16	0	0	0.24	0	0.43
Super Bright 436	0	0.97	0.09	0.07	0	0	0.86	0.01	0.22	0.06	0.01	0	0.01	0	1	0.01	0	0.09	0.03	0	0.16	0	0	0.01	0	0.09	0.03	0.05	0	0	0	0.1	0.36	0	0	0
BUV563	0.01	0.01	0.14	0	0.32	0.01	0.01	0.03	0.22	0.2	0.51	0.01	0.12	0	0.01	1	0.06	0.04	0.15	0	0.37	0.03	0.07	0.04	0.07	0.01	0.05	0	0.02	0.01	0.01	0.29	0.07	0.01	0.04	0
BUV661	0.5	0	0.04	0.05	0.11	0.71	0	0.39	0.04	0.17	0.03	0.05	0.17	0.1	0	0.06	1	0.4	0.01	0.16	0.06	0.78	0.42	0.38	0	0.25	0.04	0.09	0.29	0.37	0.65	0.02	0.01	0.1	0	0.21
BV650	0.18	0.09	0.25	0.15	0.14	0.18	0.08	0.38	0.16	0.53	0.05	0.04	0.56	0.05	0.09	0.04	0.4	1	0.06	0.07	0.25	0.37	0.29	0.38	0	0.45	0	0.23	0.31	0.13	0.19	0.02	0.06	0.02	0	0.09
Spark Blue 550	0	0.02	0.33	0.01	0.16	0	0.05	0.06	0.3	0.16	0.24	0.01	0.11	0	0.03	0.15	0.01	0.06	1	0	0.24	0.01	0.06	0.06	0.77	0.02	0.02	0.01	0.05	0.01	0	0.09	0.22	0	0.53	0.01
APC-eFluor 780	0.32	0	0	0.22	0.01	0.2	0	0.15	0	0.02	0	0.3	0.02	0.75	0	0	0.16	0.07	0	1	0.01	0.2	0.09	0.05	0	0.18	0	0.18	0.18	0.31	0.27	0	0	0.2	0	0.78
BV570	0.02	0.15	0.77	0.03	0.35	0.01	0.15	0.06	0.54	0.7	0.52	0.01	0.53	0	0.16	0.37	0.06	0.25	0.24	0.01	1	0.05	0.09	0.06	0.02	0.07	0.01	0.04	0.05	0.02	0.01	0.09	0.26	0	0.01	0.01
APC	0.57	0	0.03	0.04	0.14	0.9	0	0.37	0.02	0.14	0.04	0.05	0.19	0.13	0	0.03	0.78	0.37	0.01	0.2	0.05	1	0.52	0.34	0	0.23	0	0.07	0.29	0.24	0.75	0	0	0.02	0	0.27
PE-Cy5	0.28	0	0.02	0.03	0.36	0.32	0	0.72	0.01	0.21	0.14	0.14	0.31	0.06	0	0.07	0.42	0.29	0.06	0.09	0.09	0.52	1	0.77	0.01	0.14	0	0.05	0.47	0.1	0.28	0	0	0.01	0.01	0.11
PerCP	0.17	0.01	0.03	0.07	0.21	0.23	0.01	0.81	0.01	0.18	0.06	0.09	0.23	0.03	0.01	0.04	0.38	0.38	0.06	0.05	0.06	0.34	0.77	1	0	0.23	0	0.13	0.46	0.15	0.21	0.01	0.01	0.03	0.01	0.09
cFluor B532	0	0	0	0	0.05	0	0	0	0.02	0.01	0.09	0	0	0	0	0.07	0	0	0.77	0	0.02	0	0.01	0	1	0	0.01	0	0	0	0	0.06	0.05	0	0.89	0
BV711	0.39	0.09	0.08	0.49	0.03	0.18	0.07	0.5	0.06	0.17	0.01	0.11	0.17	0.12	0.09	0.01	0.25	0.45	0.02	0.18	0.07	0.23	0.14	0.23	0	1	0	0.69	0.65	0.41	0.27	0	0.03	0.09	0	0.32
BUV395	0	0.05	0.01	0	0.01	0	0.01	0	0.02	0.01	0.01	0	0.01	0	0.03	0.05	0.04	0	0.02	0	0.01	0	0	0	0.01	0	1	0	0	0.03	0	0.25	0.02	0.11	0.01	0
BV750	0.11	0.05	0.05	0.82	0.01	0.02	0.04	0.28	0.04	0.1	0	0.15	0.09	0.12	0.05	0	0.09	0.23	0.01	0.18	0.04	0.07	0.05	0.13	0	0.69	0	1	0.38	0.37	0.06	0	0.02	0.14	0	0.31
PerCP-eFluor 710	0.49	0	0.04	0.26	0.11	0.23	0	0.86	0.02	0.14	0.03	0.29	0.16	0.13	0	0.02	0.29	0.31	0.05	0.18	0.05	0.29	0.47	0.46	0	0.65	0	0.38	1	0.39	0.31	0	0	0.07	0	0.34
BUV737	0.45	0	0.02	0.25	0.01	0.22	0	0.31	0.02	0.05	0	0.14	0.04	0.21	0	0.01	0.37	0.13	0.01	0.31	0.02	0.24	0.1	0.15	0	0.41	0.03	0.37	0.39	1	0.28	0.01	0	0.39	0	0.53
Spark NIR 685	0.78	0	0.01	0.04	0.03	0.92	0	0.35	0	0.03	0.01	0.04	0.06	0.16	0	0.01	0.65	0.19	0	0.27	0.01	0.75	0.28	0.21	0	0.27	0	0.06	0.31	0.28	1	0	0	0.03	0	0.36
BUV496	0	0.09	0.22	0	0.01	0	0.13	0.01	0.48	0.05	0.03	0	0.02	0	0.1	0.29	0.02	0.02	0.09	0	0.09	0	0	0.01	0.06	0	0.25	0	0	0.01	0	1	0.38	0.03	0.07	0.01
BV480	0	0.28	0.58	0.02	0.03	0	0.58	0.01	0.84	0.16	0.05	0	0.1	0	0.36	0.07	0.01	0.06	0.22	0	0.26	0	0	0.01	0.05	0.03	0.02	0.02	0	0	0	0.38	1	0	0.06	0
BUV805	0.04	0.01	0	0.21	0	0.02	0	0.06	0	0.01	0	0.08	0	0.24	0	0.01	0.1	0.02	0	0.2	0	0.02	0.01	0.03	0	0.09	0.11	0.14	0.07	0.39	0.03	0.03	0	1	0	0.14
BB515	0	0	0	0	0.03	0	0	0.01	0.01	0	0.05	0	0	0	0	0.04	0	0	0.53	0	0.01	0	0.01	0.01	0.89	0	0.01	0	0	0	0	0.07	0.06	0	1	0
Zombie NIR	0.51	0	0.01	0.27	0.01	0.27	0	0.25	0.01	0.02	0	0.26	0.02	0.43	0	0	0.21	0.09	0.01	0.78	0.01	0.27	0.11	0.09	0	0.32	0	0.31	0.34	0.53	0.36	0.01	0	0.14	0	1

Complexity™ Index: 37.73

Supplementary Figure S2. Panel similarity and complexity indices. The Similarity™ Index calculates how similar two spectra are to one other. A value of 1 equates to no difference between two fluorochromes, whereas a value of 0 indicates that two fluorochromes signatures are entirely different. Each pair of fluorochromes identified for use in the panel is shown numerically in the chart. Based on several fluorochrome combinations tested in the OMIP-069 study (Park *et al.*, 2020), it was established that any fluorochrome pair with a similarity index of 0.98 or below could be accurately unmixed with adequate single stained controls. The Complexity™ Index is 37.73.
