# Supplementary material for: Deep immune profiling of endometrial and peripheral blood cells in endometriosis
Source: Hum Reprod. 2026 Jun 5;41(8):1324–37. doi: 10.1093/humrep/deag090 (PMC13429876; doi:10.1093/humrep/deag090)
Supplement: deag090_Supplementary_Figure_S3 [file deag090_supplementary_figure_s3.pptx]

## Slide 1
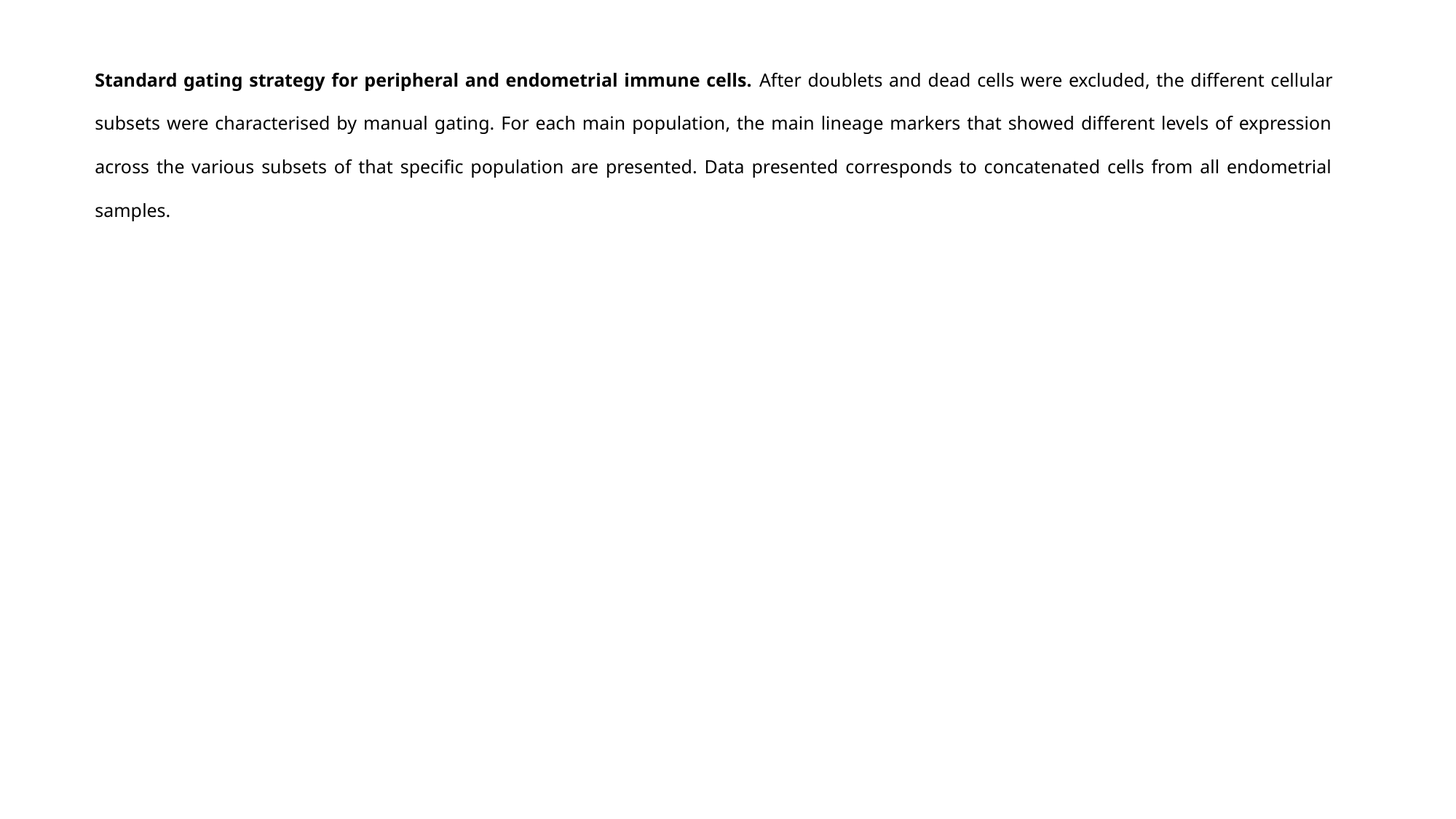

Standard gating strategy for peripheral and endometrial immune cells. After doublets and dead cells were excluded, the different cellular subsets were characterised by manual gating. For each main population, the main lineage markers that showed different levels of expression across the various subsets of that specific population are presented. Data presented corresponds to concatenated cells from all endometrial samples.

## Slide 2
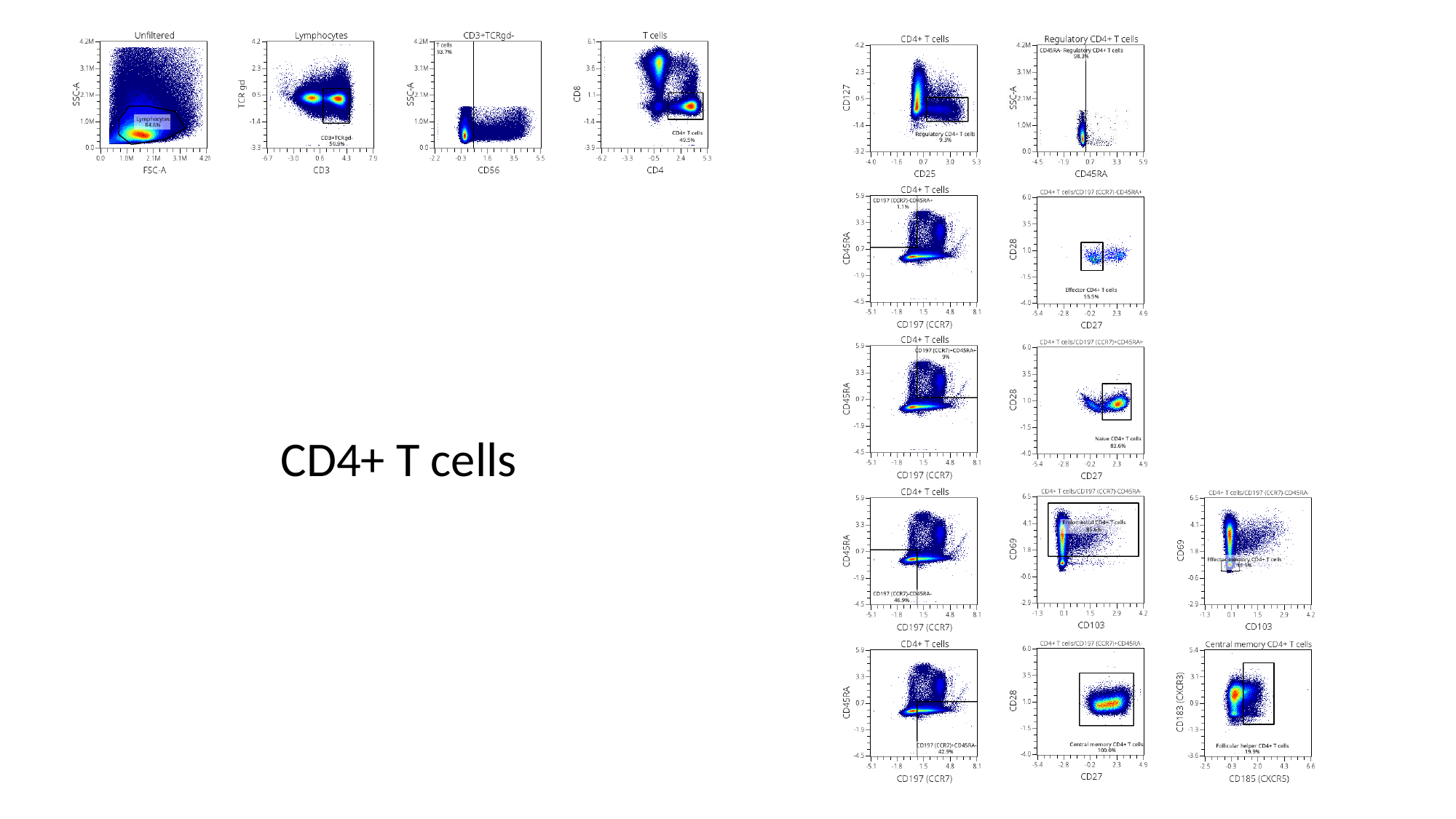

CD4+ T cells

## Slide 3
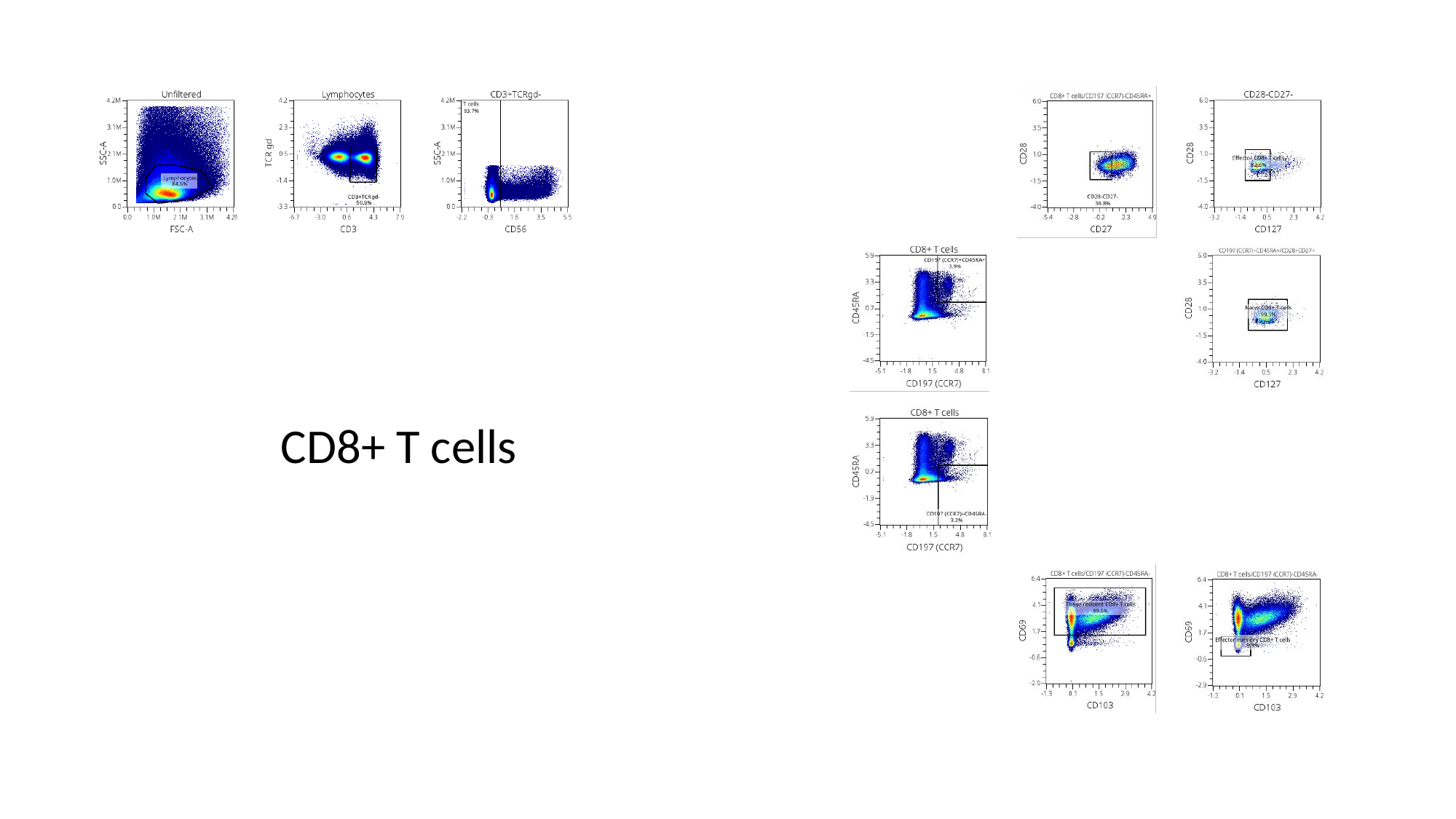

CD8+ T cells

## Slide 4
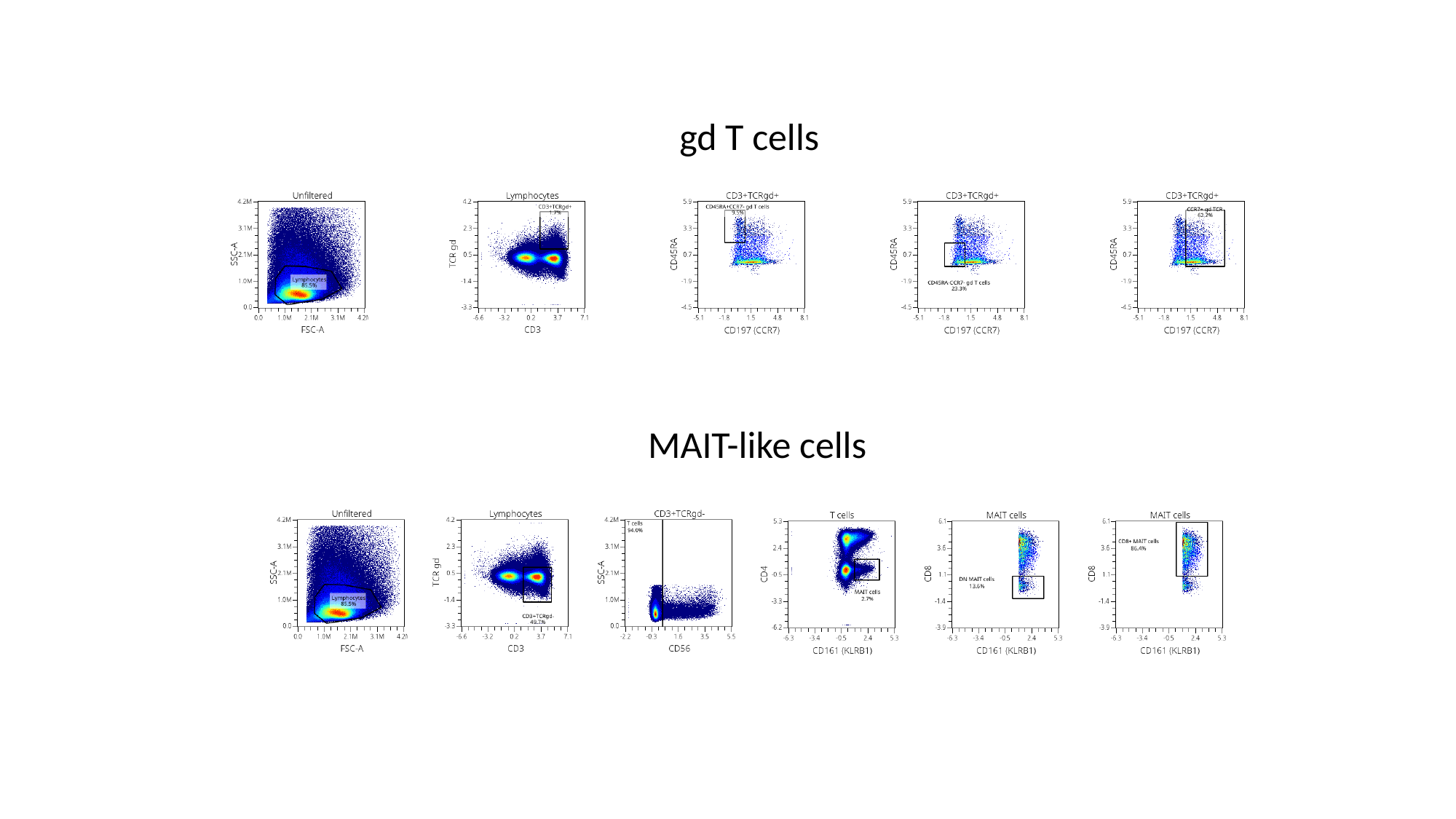

gd T cells
MAIT-like cells

## Slide 5
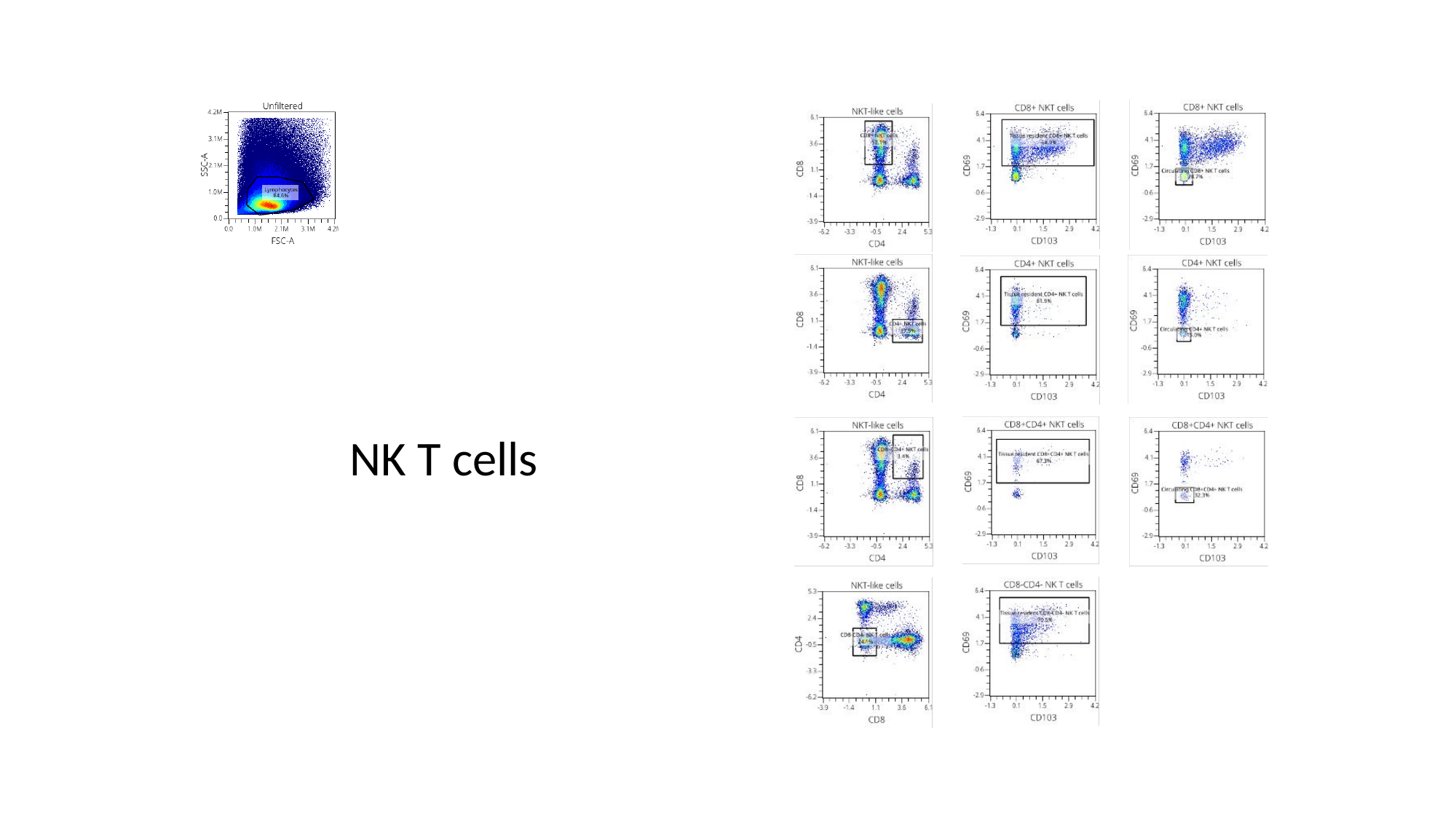

NK T cells

## Slide 6
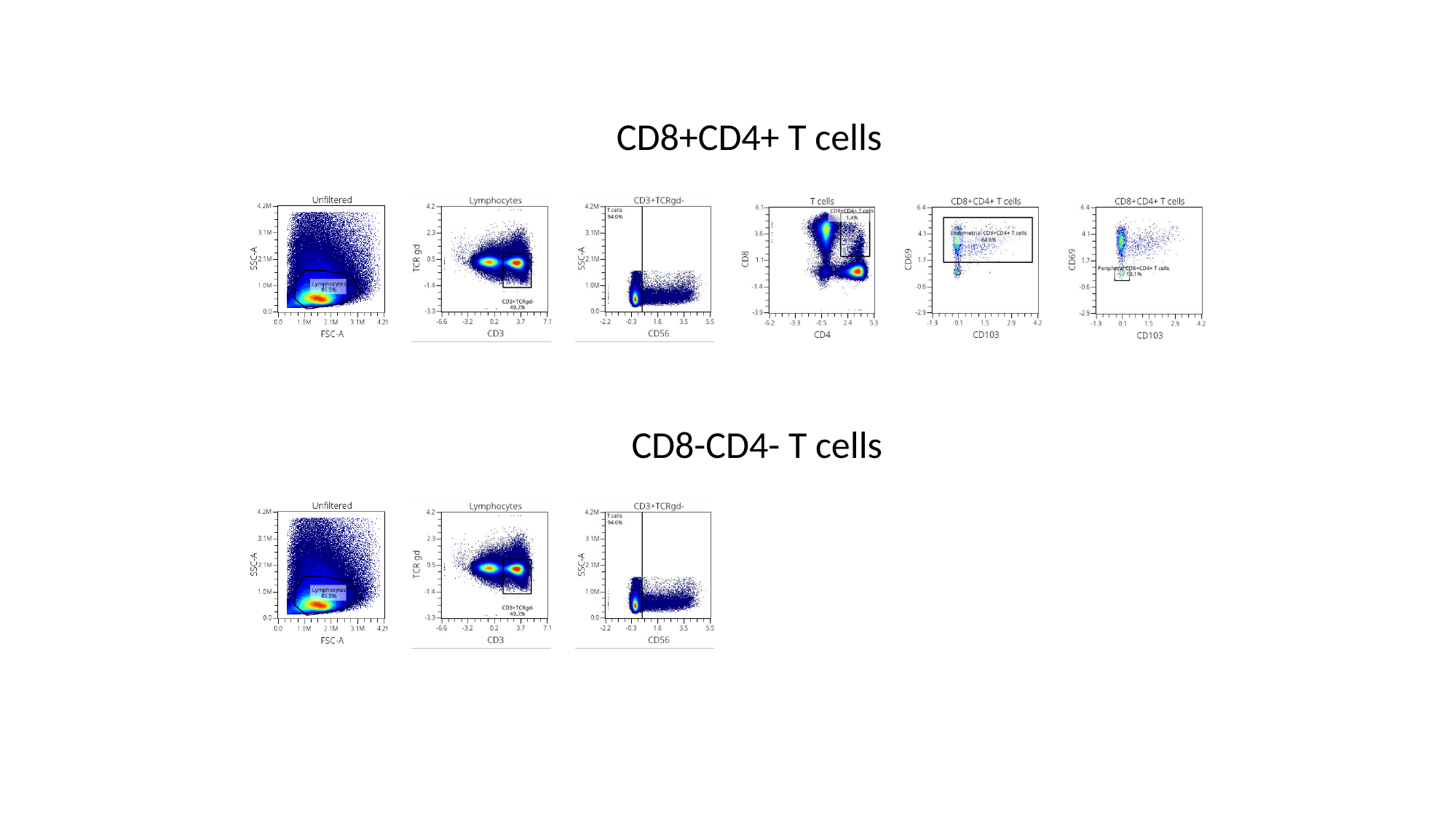

CD8+CD4+ T cells
CD8-CD4- T cells

## Slide 7
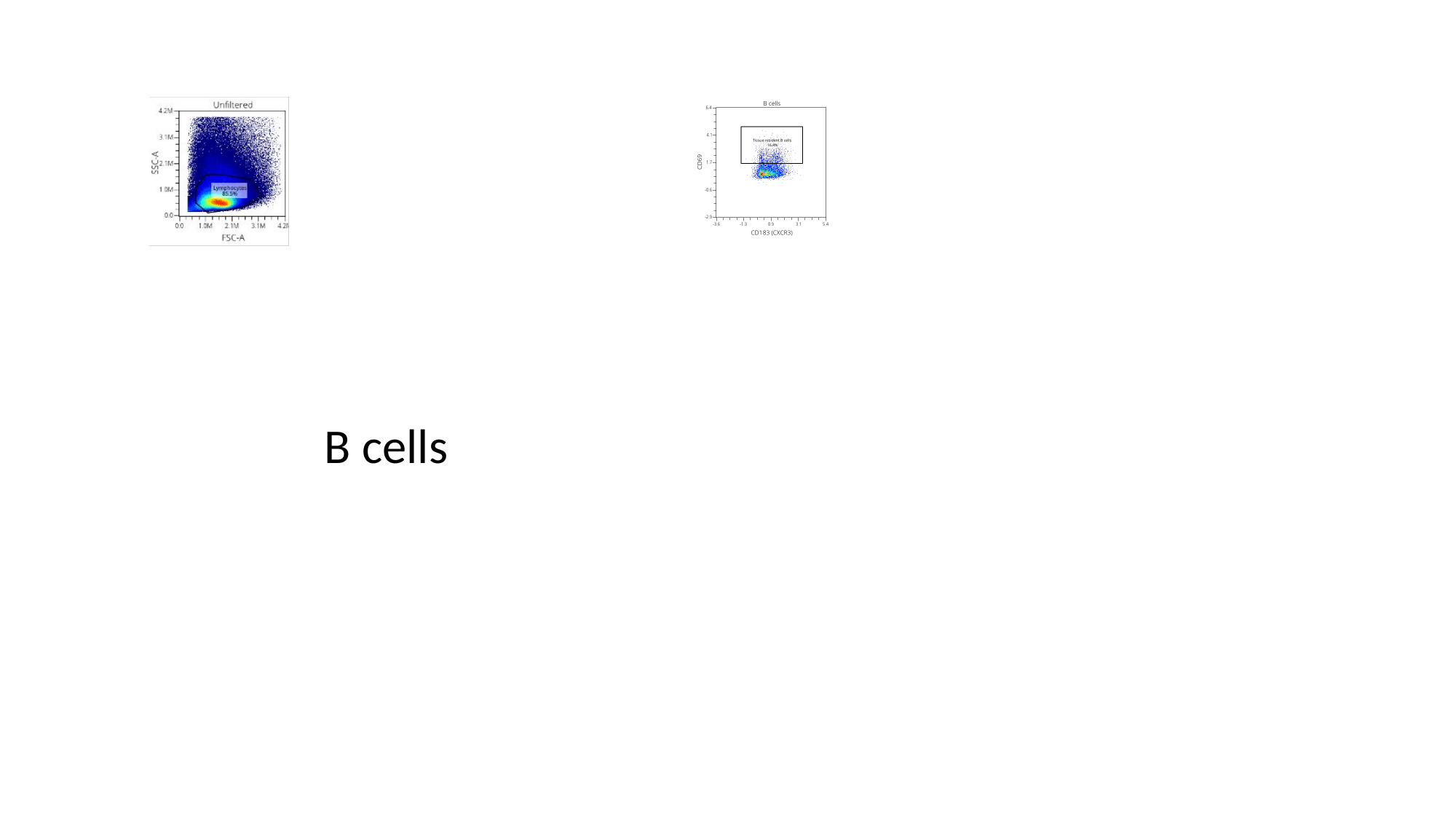

B cells

## Slide 8
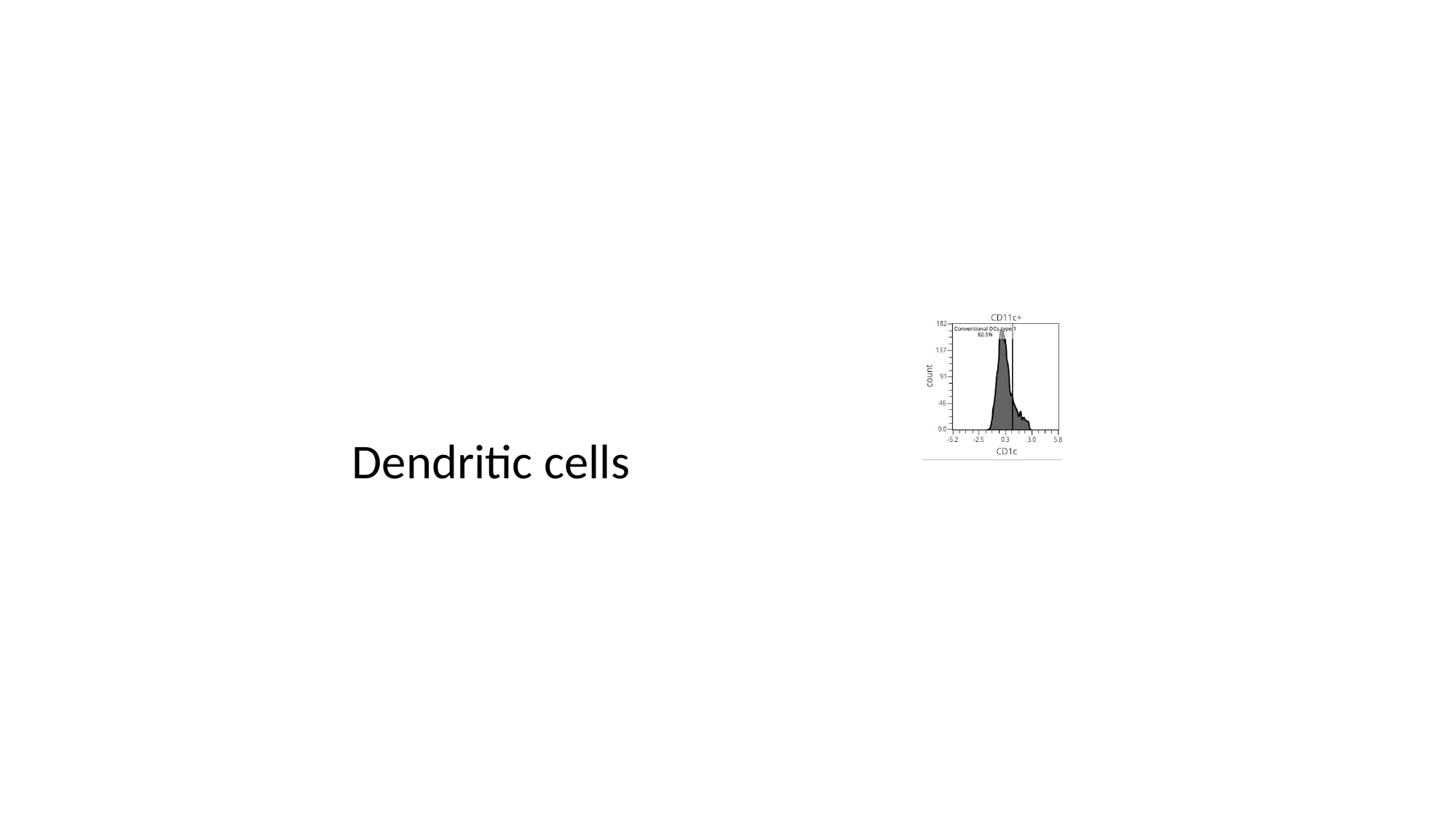

Dendritic cells

## Slide 9
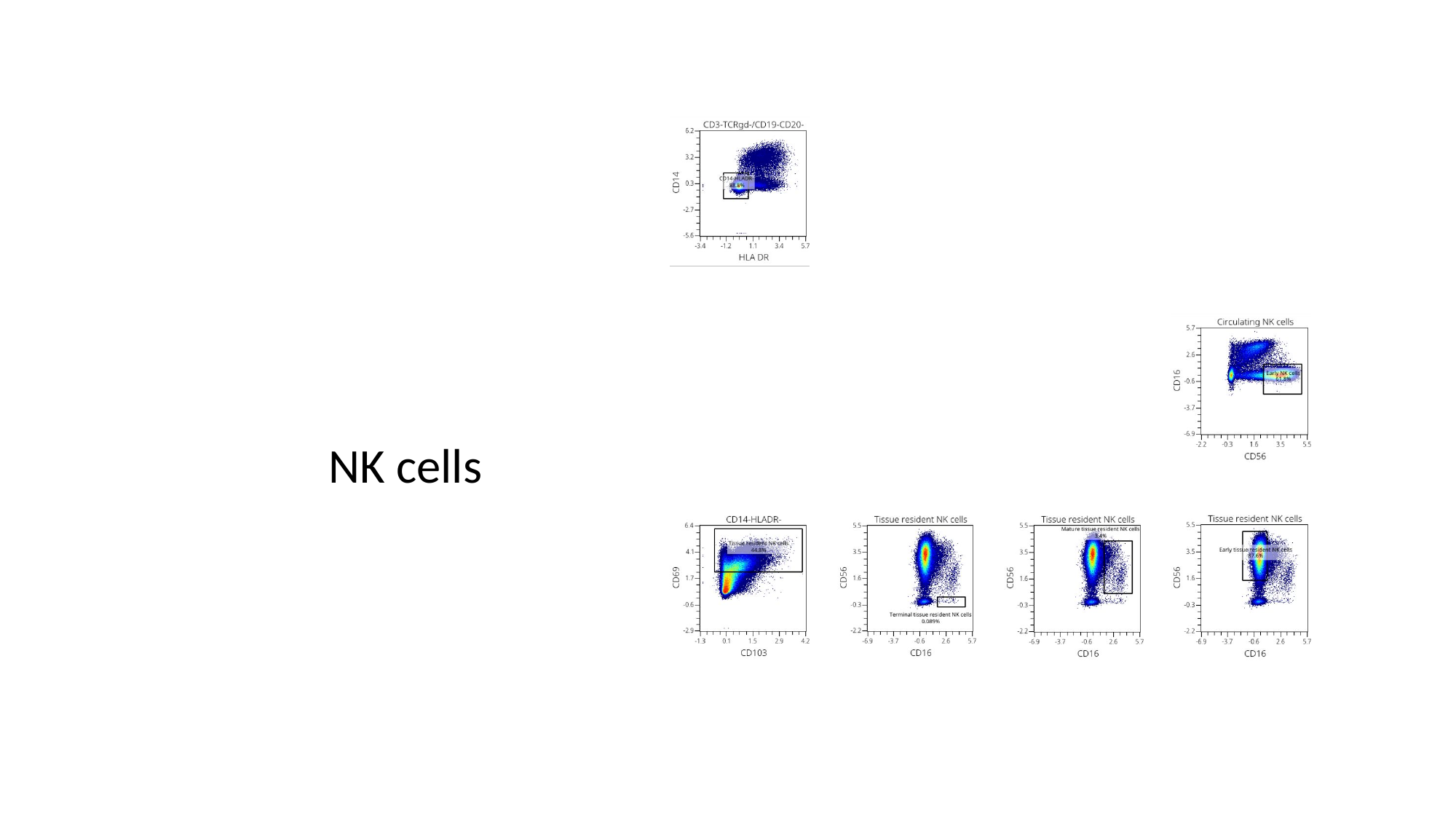

NK cells

## Slide 10
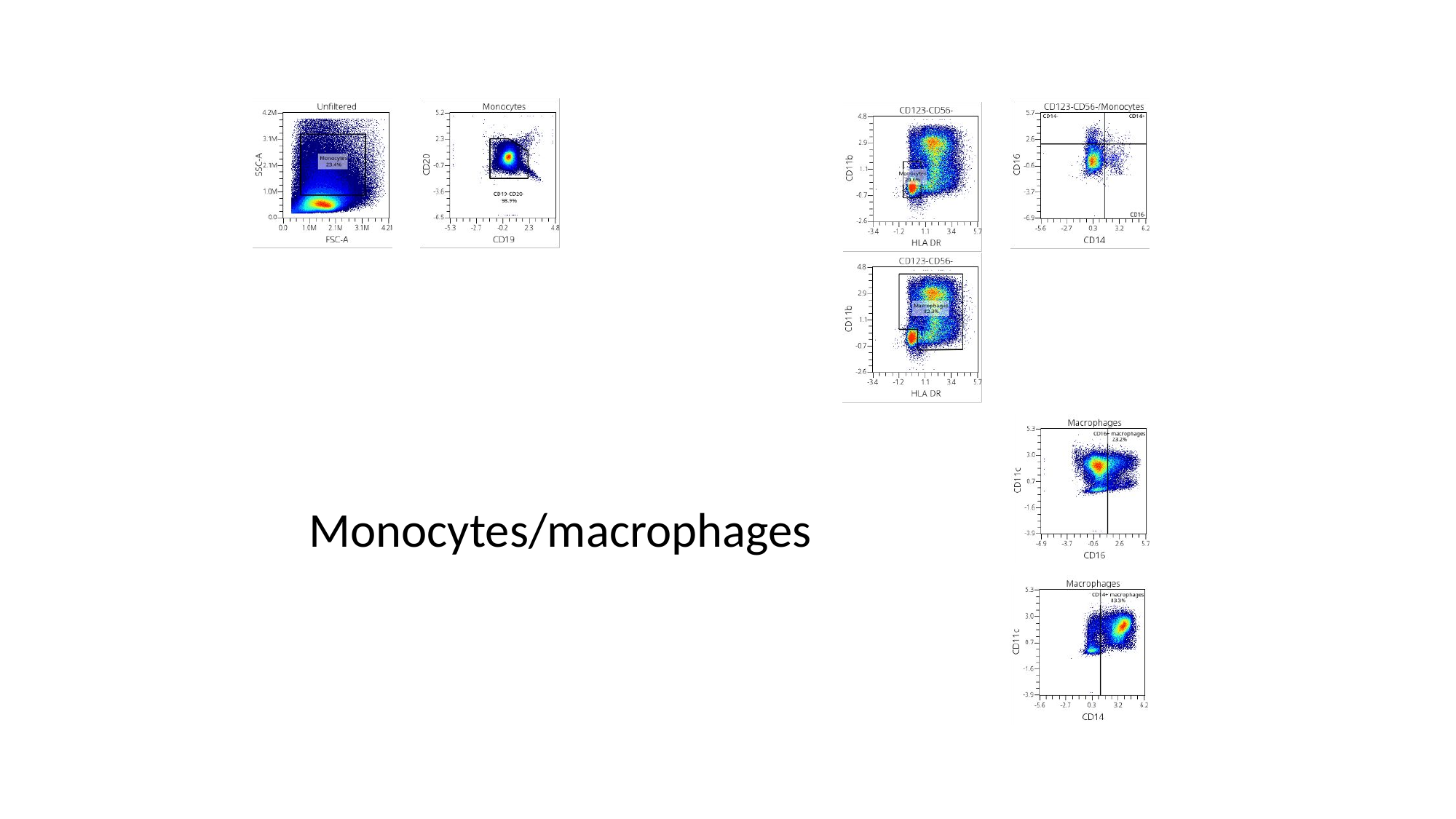

Monocytes/macrophages

## Slide 11
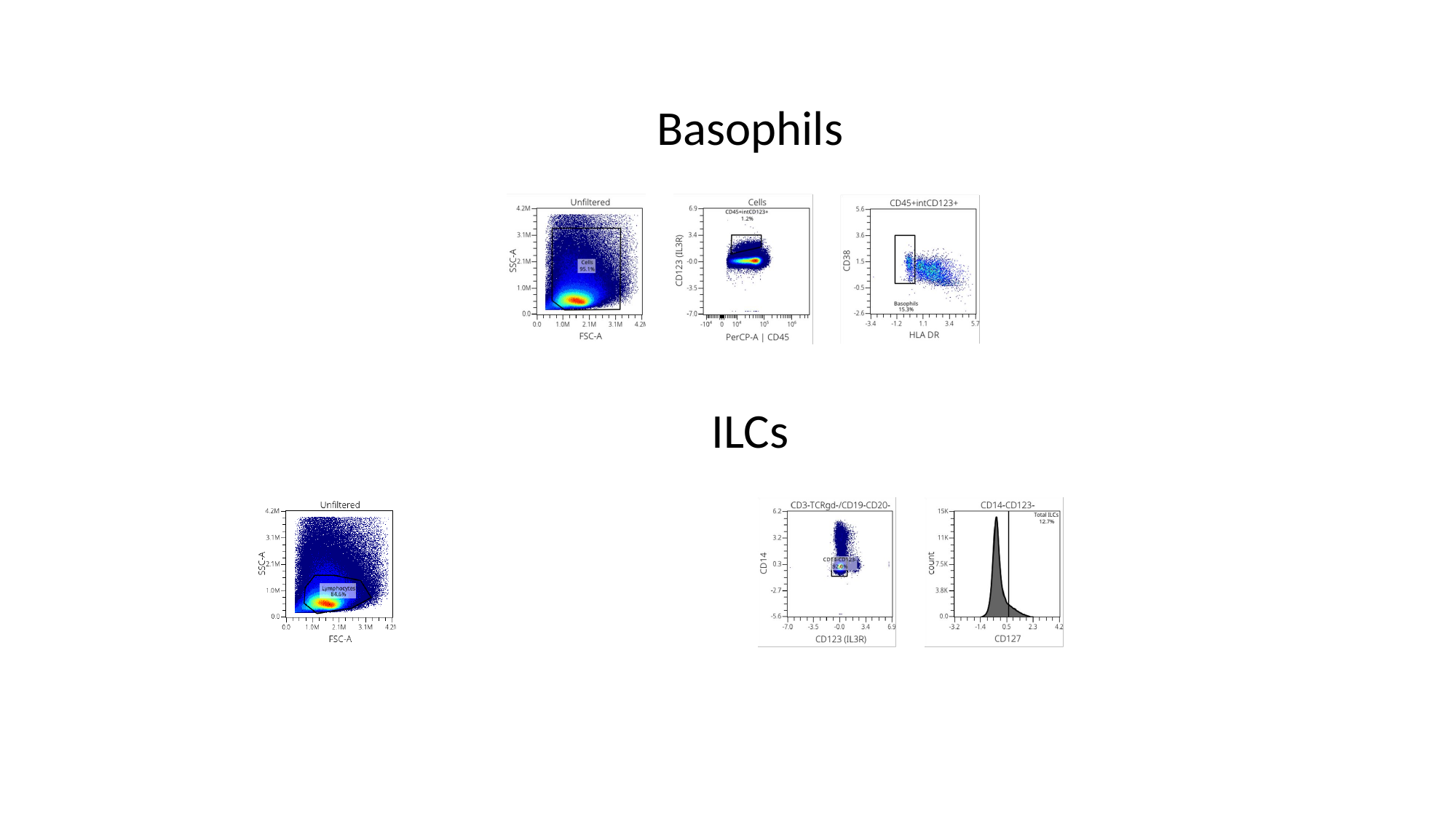

Basophils
ILCs
