# Supplementary material for: Deep immune profiling of endometrial and peripheral blood cells in endometriosis
Source: Hum Reprod. 2026 Jun 5;41(8):1324–37. doi: 10.1093/humrep/deag090 (PMC13429876; doi:10.1093/humrep/deag090)
Supplement: deag090_Supplementary_Figure_S4 [file deag090_supplementary_figure_s4.pdf]

Human Reproduction, 2026, 1–1
https://doi.org/10.1093/humrep/deag090
Supplementary Data

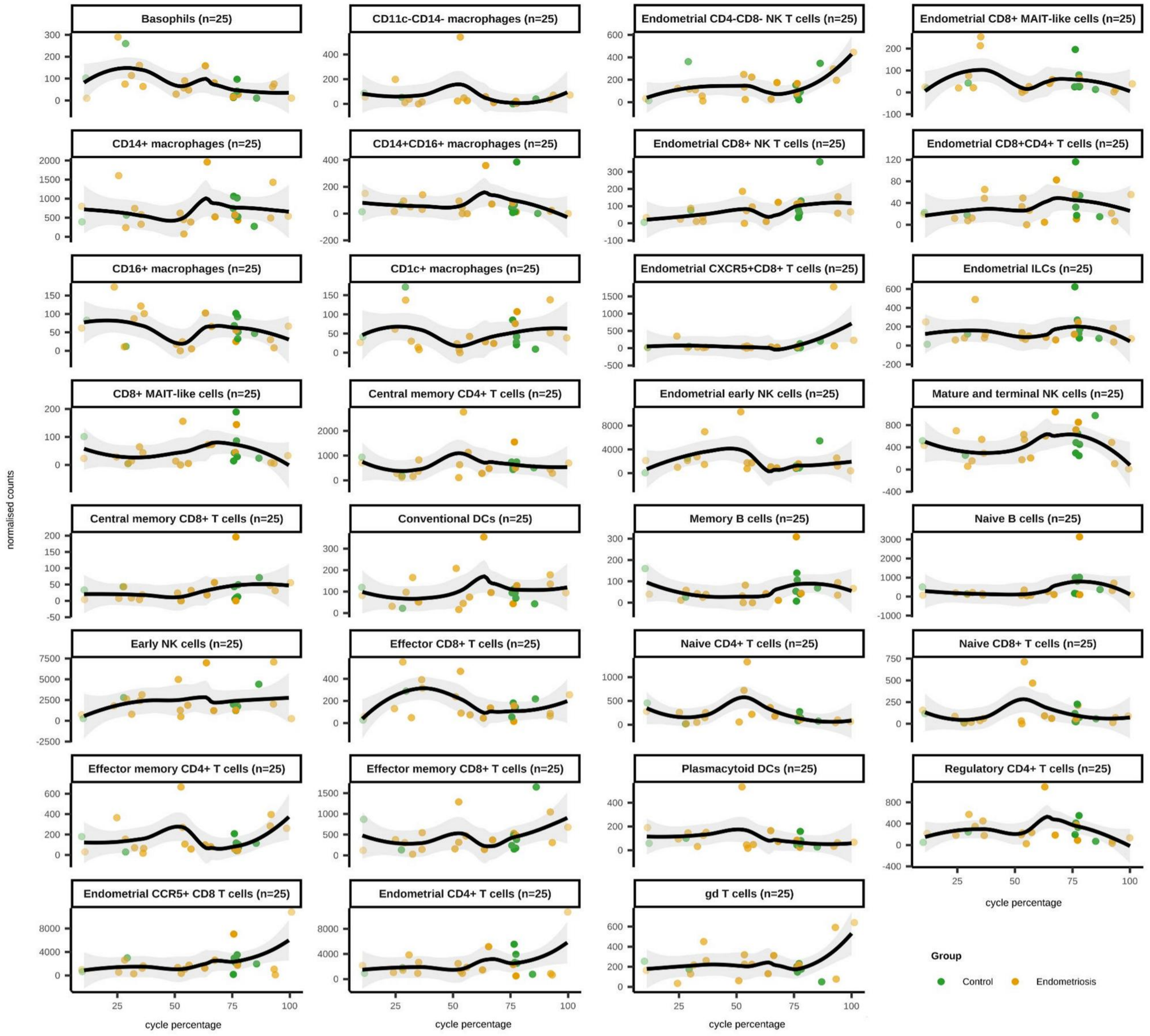

Supplementary Figure S4. **Endometrial immune cell counts across the menstrual cycle.** Raw data of normalized cell counts of clusters across the menstrual cycle stage for the 31 immune cell clusters identified through spectral flow cytometric analysis of endometrial samples, showing individual data points for control (green dots, n = 8) and endometriosis (orange dots, n = 17) with confidence interval (grey shade). For each cluster, normalized counts were plotted against cycle percentage and trends were estimated using LOESS smoothing, with 95% confidence intervals visualized as shaded ribbons. Points represent individual group-specific samples (endometriosis in orange and control in green), jittered along the *x*-axis, with transparency scaled by local sample density.
