# Supplementary material for: Deep immune profiling of endometrial and peripheral blood cells in endometriosis
Source: Hum Reprod. 2026 Jun 5;41(8):1324–37. doi: 10.1093/humrep/deag090 (PMC13429876; doi:10.1093/humrep/deag090)
Supplement: deag090_Supplementary_Figure_S5 [file deag090_supplementary_figure_s5.pdf]

Human Reproduction, 2026, 1–1
https://doi.org/10.1093/humrep/deag090
Supplementary Data

human reproduction

OXFORD

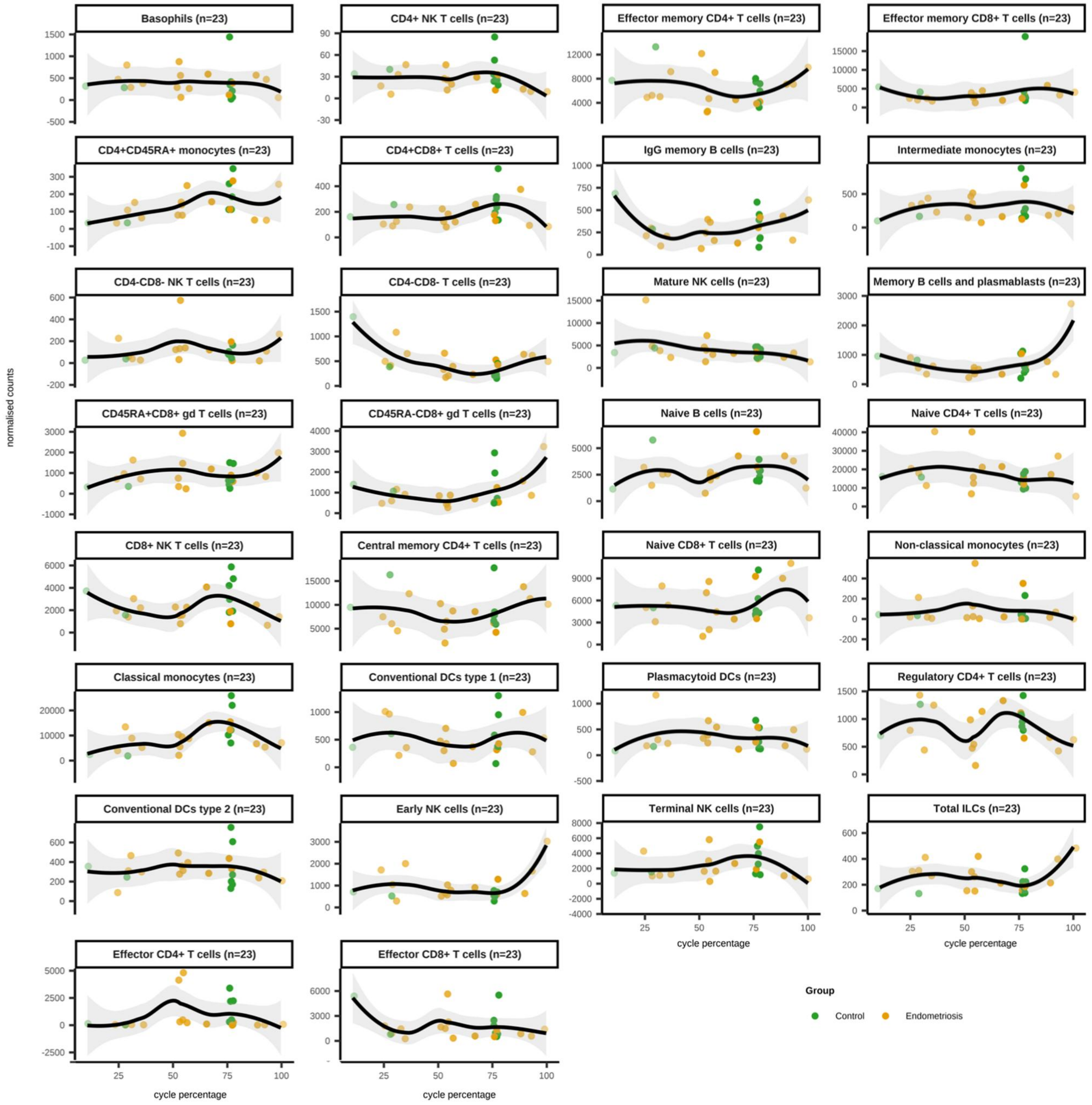

Supplementary Figure S5. Peripheral blood immune cell counts across the menstrual cycle. Raw data of normalized cell counts detected in peripheral blood samples versus menstrual cycle stage for the 30 immune cell clusters identified through spectral flow cytometry, showing individual data points for control (green dots, n = 8) and endometriosis (orange dots, n = 15) with confidence interval (grey shade). For each cluster, normalized counts were plotted against cycle percentage and trends were estimated using LOESS smoothing, with 95% confidence intervals visualized as shaded ribbons. Points represent individual group-specific samples (endometriosis in orange and control in green), jittered along the *x*-axis, with transparency scaled by local sample density.
