# Supplementary material for: Deep immune profiling of endometrial and peripheral blood cells in endometriosis
Source: Hum Reprod. 2026 Jun 5;41(8):1324–37. doi: 10.1093/humrep/deag090 (PMC13429876; doi:10.1093/humrep/deag090)
Supplement: deag090_Supplementary_Figure_S6 [file deag090_supplementary_figure_s6.pdf]

Human Reproduction, 2026, 1–1
https://doi.org/10.1093/humrep/deag090
Supplementary Data

human reproduction
OXFORD

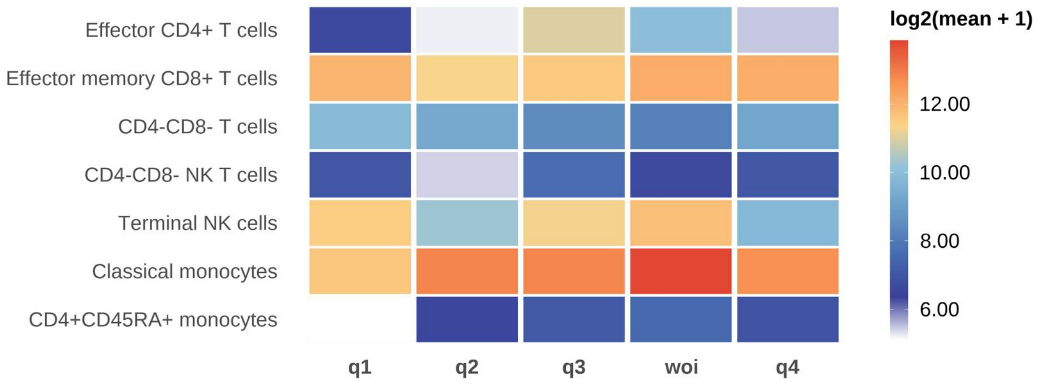

Supplementary Figure S6. Significant changes to peripheral blood immune cell counts across the menstrual cycle. Heat map depicting changes in cell counts across the menstrual cycle for significantly altered populations; data comprised all endometriosis (n = 15) and control (n = 8) patients. Menstrual cycle was divided into four menstrual cycle quarters and window of implantation (WOI); cell counts detected at each stage are shown; P.adj < 0.05 and $|\log2FC| > 1$ were applied.
