# Supplementary material for: Deep immune profiling of endometrial and peripheral blood cells in endometriosis
Source: Hum Reprod. 2026 Jun 5;41(8):1324–37. doi: 10.1093/humrep/deag090 (PMC13429876; doi:10.1093/humrep/deag090)
Supplement: deag090_Supplementary_Table_S1 [file deag090_supplementary_table_s1.pdf]

Human Reproduction, 2026, 1–1
https://doi.org/10.1093/humrep/deag090
Supplementary Data

human reproduction

OXFORD

Supplementary Table S1. Reagents used for the deep immune phenotyping with full-spectrum flow cytometry.

Marker	Clone	Per test (μl)	Lot number	Fluorochrome
**Viability**				Zombie NIR
**CD45**	HI130	2	B331250	PerCP
**CD3**	SK7	5	B330335, B370758	BV510
**CD4**	SK3	2	F-050721-01	cFluor® B532
**CD8**	SK1	5	n/a	cFluor® V610
**CD25**	BC96	2	B354215	PE-Cy5
**TCRγδ**	B1.1	1.2	2311939, 2527383	PerCP-eFluor 710
**CD14**	63D3	2.5	B313132, B329322	Spark Blue 550
**CD16**	3G8	0.6	1099493	BUV496
**CD11c**	B-ly6	2.5	1148605, 2111332	BUV661
**CD11b**	ICRF44	5	B319299	PerCP-Cy5.5
**CD19**	HIB19	1.2	B324542	Spark NIR 685
**CD20**	HI47	5	2375623, 2532779	Pacific Orange
**CD24**	SN3	5	2363760, 2412844	PE-eFluor 610
**IgD**	IA6-2	0.6	1195093	BV480
**IgG**	G18.145	5	300956, 1292726, 2052950	BV605
**IgM**	MHM-88	2.5	B316253, B334624	BV570
**CD1c**	L161	5	B293062	Alexa Fluor 647
**CD123 (IL3R)**	6H6	2.5	2305276	Super Bright 436
**CD56**	NCAM16.2	1.2	1048151	BUV737
**CD197 (CCR7)**	G043H7	5	B332066, B361376	BV421
**CD27**	O323	2.5	2288534, 2443439	APC
**CD28**	CD28.2	2.5	B324232	BV650
**CD45RA**	5H9	1.2	1210597	BUV395
**CD127**	HIL-7R-M21	6	1208740, 1341730, 2292425	APC-R700
**CD196 (CCR6)**	g034e3	1.25	B309645, B369286	BV711
**CD195 (CCR5)**	2D7/CCR5	2.5	1210625, 3051963	BUV563
**CD185 (CXCR5)**	RF8B2	1.2	1210649	BV750
**CD183 (CXCR3)**	G025H7	5	B354664	PE
**HLA DR**	L243	2.5	2406121	APC-eFluor 780
**CD38**	HIT2	3	B325373	APC/Fire 810
**CD57**	NK-1	0.2	1273657	BB515
**CD161 (KLRB1)**	HP-3G10	5	2480316	eFluor 450
**CD279 (PD1)**	EH12.2H7	5	B318729, B373725	BV785
**CD69**	FN50	5	B350377	PE-Cy7
**CD103**	Ber-ACT8	0.5	2236472	BUV805
