# Supplementary material for: Deep immune profiling of endometrial and peripheral blood cells in endometriosis
Source: Hum Reprod. 2026 Jun 5;41(8):1324–37. doi: 10.1093/humrep/deag090 (PMC13429876; doi:10.1093/humrep/deag090)
Supplement: deag090_Supplementary_Table_S2 [file deag090_supplementary_table_s2.pdf]

Human Reproduction, 2026, 1–1
https://doi.org/10.1093/humrep/deag090
Supplementary Data

Supplementary Table S2. Patient metadata for participants in the exploratory and case: control cohort.

Number	Age	BMI	Cycle quarter	Percentage of completed cycle	Endometriosis	Stage	Parity	Miscarriage	Miscarriage >3	Subfertility	Next pregnancy outcome	Case: control study
1	37	39	1	11	Yes	2	Yes	NA	No	Yes	NA	Endometriosis
2	32	27	1	24	Yes	1	NA	NA	NA	NA	NA	Excluded
3	34	25	1	25	Yes	3	No	No	No	NA	NA	Endometriosis
4	40	NA	2	29	Yes	1	Yes	No	No	No	NA	Endometriosis
5	23	19	2	32	Yes	1	No	No	No	NA	NA	Endometriosis
6	44	26	2	36	Yes	4	No	No	No	NA	NA	Endometriosis
7	28	22	2	36	Yes	1	Yes	Yes	No	No	NA	Endometriosis
8	38	20	3	52	Yes	2	Yes	No	No	Yes	NA	Endometriosis
9	26	30	3	54	Yes	3	No	No	No	NA	NA	Endometriosis
10	34	29	3	54	Yes	1	Yes	Yes	No	No	NA	Endometriosis
11	34	21	3	54	Yes	3	No	No	No	NA	NA	Endometriosis
12	36	19	3	57	Yes	3	Yes	Yes	No	Yes	NA	Endometriosis
13	29	20	3	64	Yes	4	No	No	No	NA	NA	Endometriosis
14	35	42	3.5	67	Yes	1	No	No	No	Yes	NA	Endometriosis
15	35	22	3.5	77	Yes	4	No	Yes	No	No	NA	Endometriosis
16	35	21	3.5	77	Yes	NA	No	Yes	No	No	Miscarriage	Endometriosis
17	30	35	4	89	Yes	1	Yes	Yes	No	No	NA	Endometriosis
18	30	NA	4	93	Yes	4	No	No	No	NA	NA	Endometriosis
19	30	32	4	93	Yes	1	Yes	No	No	No	NA	Endometriosis
20	34	20	4	100	Yes	2	No	No	No	NA	NA	Endometriosis
21	18	24	1	11	No	NA	No	No	No	NA	NA	Control
22	33	36	2	29	No	NA	No	No	No	NA	NA	Control
23	35	39	3.5	77	No	NA	No	Yes	Yes	No	Miscarriage	Excluded
24	34	24	3.5	77	No	NA	No	Yes	Yes	No	Miscarriage	Excluded
25	33	26	3.5	77	No	NA	No	Yes	No	No	Live birth	Control
26	32	31	3.5	77	No	NA	Yes	Yes	No	No	Live birth	Control
27	37	23	3.5	77	No	NA	No	Yes	No	No	Live birth	Control
28	30	30	3.5	77	No	NA	No	Yes	Yes	No	Live birth	Excluded
29	36	33	3.5	77	No	NA	No	Yes	No	No	Miscarriage	Control
30	34	31	3.5	77	No	NA	Yes	Yes	No	No	Miscarriage	Control
31	36	21	3.5	77	No	NA	Yes	Yes	Yes	No	Live birth	Excluded
32	33	30	3.5	86	No	NA	No	No	No	NA	NA	Control
