# Supplementary material for: Deep immune profiling of endometrial and peripheral blood cells in endometriosis
Source: Hum Reprod. 2026 Jun 5;41(8):1324–37. doi: 10.1093/humrep/deag090 (PMC13429876; doi:10.1093/humrep/deag090)
Supplement: deag090_Supplementary_Table_S3 [file deag090_supplementary_table_s3.pdf]

Human Reproduction, 2026, 1–1
https://doi.org/10.1093/humrep/deag090
Supplementary Data

human reproduction

OXFORD

Supplementary Table S3. Patient metadata for participants in the validation cohort.

Number	Age	BMI	Cycle quarter	Percentage of completed cycle	Endometriosis stage	Subfertility
1	41	22.9	1	18	2	No
2	22	20.07	2	48	2	No
3	42	21.9	1	13	2	Yes
4	44	22.86	4	79	NA	No
5	27	29.56	3.5	75	NA	No
6	41	19.81	3	71	1	No
7	40	35.69	4	97	1	Yes
8	34	20	3.5	75	NA	No
