# Supplementary material for: Deep immune profiling of endometrial and peripheral blood cells in endometriosis
Source: Hum Reprod. 2026 Jun 5;41(8):1324–37. doi: 10.1093/humrep/deag090 (PMC13429876; doi:10.1093/humrep/deag090)
Supplement: deag090_Supplementary_Table_S4 [file deag090_supplementary_table_s4.pdf]

Human Reproduction, 2026, 1–1
https://doi.org/10.1093/humrep/deag090
Supplementary Data
human reproduction
OXFORD

Supplementary Table S4. Patient metadata for participants in the imaging cohort.

Number	Age	BMI	Cycle quarter	Percentage of completed cycle	Endometriosis	Parity	Miscarriage	Contraception	Symptoms	Histology
1	34	NA	4	100	No	Yes	No	IUD	Menorrhagia, dysmenorrhoea	Late secretory phase endometrium, some inflammatory debris within glands, could be due to IUCD. No hyperplasia or malignancy
2	27	NA	1	7	Yes	No	Yes	Oral contraception	Menorrhagia, dysmenorrhoea, occasional bowel pain	Endometrium is mainly basal and predominantly inactive. No evidence of chronic endometritis, polyp formation, hyperplasia, atypia, or malignancy
3	44	34	2	36	Yes	No	No	None	Subfertility	Proliferative
